# Mitigating Cell Cycle Effects in Multi‐Omics Data: Solutions and Analytical Frameworks

**DOI:** 10.1002/advs.202505823

**Published:** 2025-05-28

**Authors:** Rui Nie, Caihong Zheng, Likun Ren, Yue Teng, Yaoyu Sun, Lifei Wang, Junya Li, Jun Cai

**Affiliations:** ^1^ Chinese Academy of Sciences and China National Center for Bioinformation Beijing 100101 China; ^2^ University of Chinese Academy of Sciences Beijing 100049 China; ^3^ Laboratory of Molecular Biology and Bovine Breeding Institute of Animal Science Chinese Academy of Agricultural Sciences Beijing 100193 China; ^4^ School of Life Sciences Peking University Beijing 100871 China; ^5^ Department of Chemistry the University of Hong Kong Hong Kong 999077 China

**Keywords:** cell cycle compositions, pseudo‐omics features, S phase ratios

## Abstract

Cell cycle structures vary significantly across cell types, which exhibit distinct phase compositions. Asynchronous DNA replication and dynamic cellular characteristics during the cell cycle result in considerable heterogeneity in DNA dosage, chromatin accessibility, methylation, and expression. Nonetheless, the consequences of cell cycle disruption in the interpretation of multi‐omics data remain unclear. Here, we systematically assessed the influence of distinct cell phase structures on the interpretation of omics features in proliferating cells, and proposed solutions for each omics dataset. For copy number variation (CNV) calling, asynchronous replication timing (RT) interference induces false CNVs in cells with high S‐phase ratio (SPR), which are significantly decreased following replication timing domain (RTD) correction. Similar noise is observed in the chromatin accessibility data. Moreover, for DNA methylation and transcriptomic analyses, cell cycle‐sorted data outperformed direct comparison in elucidating the biological features of compared cells. Additionally, we established an integrated pipeline to identify differentially expressed genes (DEGs) after cell cycle phasing. Consequently, our study demonstrated extensive cell‐cycle heterogeneity, warranting consideration in future studies involving cells with diverse cell‐cycle structures. RTD correction or phase‐specific comparison could reduce the influence of cell cycle composition on the analysis of the differences observed between stem and differentiated cells.

## Graphical abstract

The study highlights the cell‐cycle effects on multi‐omics analysis attributed to varying cell cycle compositions across cell types. To minimize the influence, we propose RTD correction in CNV calling with a high S‐phase ratio, and phase comparison in chromatin accessibility, DNA methylation, and transcriptomics. These solutions enhance differential analysis between differentiated and proliferating cells, particularly in tumor and stem cells.

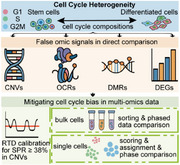

## 1 Introduction

The structure of the cell cycle phases varies among different cell types. Mammalian pluripotent stem cells (PSCs) typically proliferate rapidly and are characterized by a higher percentage of cells in the S phase than differentiated cells.^[^ ^1^, ^2^, ^3^ ^]^ During the S phase, asynchronous DNA replication results in unequal DNA dosages.^[^ ^4^, ^5^ ^]^ Additionally, there is a prolonged delay in the reestablishment of methylation after DNA synthesis.^[^ ^6^, ^7^ ^]^ Furthermore, the chromatin structure undergoes significant changes throughout the cell cycle, accompanied by alterations in transcriptomic and epigenetic patterns. Consequently, extensive heterogeneity has been observed in DNA dosage, chromatin accessibility, DNA methylation, and transcriptomes across different cell cycle stages.^[^ ^8^, ^9^, ^10^, ^11^, ^12^, ^13^, ^14^ ^]^

**Figure 1:**
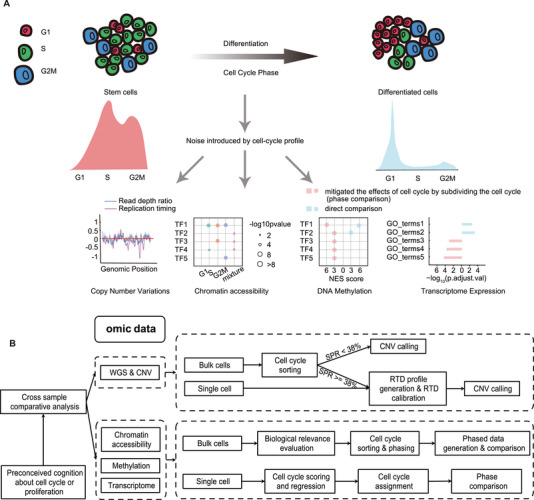
Schematic overview of the influence of cell cycle composition. A) Omics difference during phase comparison of samples, such as stem and somatic cell populations, when distinguishing the cell cycle in copy number variation, chromatin accessibility, DNA methylation, and transcriptome expression. B) The workflow for phase comparison.

Cell cycle heterogeneity exerts an influence on the interpretation of omics data.^[^ ^15^, ^16^, ^17^ ^]^ The presence of asynchronous DNA significantly affects the detection of copy number variations (CNVs) based on read depth.^[^ ^4^, ^15^, ^18^, ^19^ ^]^ Notably, the results of single‐cell genomics underscore the variability in the DNA replication status,^[^ ^20^ ^]^ and the organization of DNA replication domains is specific to each cell type at the single‐cell level.^[^ ^19^, ^21^, ^22^ ^]^ Profiling of replication timing (RT), which delineates the temporal order of replication across various segments in relation to their population average replication time,^[^ ^23^, ^24^ ^]^ yields false‐positive results attributable to the copy number profiling of cells in the S‐phase.^[^ ^20^, ^25^ ^]^ Approximately 5–20% of S‐phase cells were deemed sufficient to accurately reflect signals in underlying DNA copy numbers emanating from replicating cells.^[^ ^13^, ^26^ ^]^ Furthermore, a comprehensive reduction in intermediate CpG methylation in highly proliferative cells has been documented due to the delayed methylation of nascent strand DNA.^[^ ^27^ ^]^ The impact of the cell cycle on omics data has been widely acknowledged.^[^ ^22^, ^28^, ^29^ ^]^ In practical terms, recalibration of the expression matrix via the expression of cell cycle‐specific genes is advantageous for cell clustering, dimensionality reduction, and cell type identification.^[^ ^30^ ^]^ Nonetheless, during the detection of differentially expressed genes(DEGs), the influence of the cell cycle is not accounted for in the prevailing single‐cell RNA sequencing (scRNA‐seq) analysis frameworks, such as MAESTRO^[^ ^31^ ^]^ and Seurat.^[^ ^32^ ^]^ Consequently, we posit that for cell types exhibiting disparate cell cycle structures, a direct comparison of omics data would encompass variations introduced by different cell cycle phases, ultimately compromising the interpretation of omics features that reflect genuine biological distinctions between cell types (**Figure** **1**A).

In this study, we comprehensively examined the impact of varying cell‐cycle phase compositions on the interpretation of omics characteristics pertinent to proliferating cells. With respect to genomic attributes, we determined the correlation of CNVs associated with the replication timing domain (RTD), which is predominantly observed in stem cells as opposed to differentiated cells. These CNVs emerged as a consequence of the prolonged S phase in stem cells and could be rectified through RTD normalization. Moreover, we evaluated the proportion of S‐phase cells that could introduce significant noise into CNV detection under conventional parameters, and the simulation data indicated that when the S‐phase ratio (SPR) exceeded 38%, there was a notable increase in false‐positive signals for CNV detection. A comparable disruption was noted in the identification of open chromatin regions (OCRs) in cells with elevated SPR using chromatin accessibility data, and false‐positive OCRs exhibited a strong correlation with pseudo‐CNVs engendered by asynchronous DNA replication. Furthermore, concerning transcriptomic and methylomic analyses, a phase‐specific comparison (referred to as “phase comparison” in the rest of the paper) of cell cycle‐segregated data between two samples with different cell cycle compositions proved to be more effective in reflecting genuine biological differences than a direct comparison of bulk omics data. Notably, for the extensively used scRNA‐seq, we developed a comprehensive pipeline capable of identifying DEGs through phase comparison of cell cycle‐divided data. Consequently, we assert that for the two groups of samples characterized by distinct cell cycle architectures, such as embryonic stem cells (ESCs) and their differentiated counterparts, the variations induced by the cell cycle must be acknowledged and addressed. For genomic datasets, it is advisable to perform calibration using RTD prior to CNV detection. For other omics data, the division of cell cycles and subsequent phase comparison were advantageous for conducting direct comparisons between samples (Figure 1B).

## 2 Results

### 2.1 Extended S Phase of Cell Cycle Introducing Pseudo CNVs in Proliferating Cells

**Figure 2:**
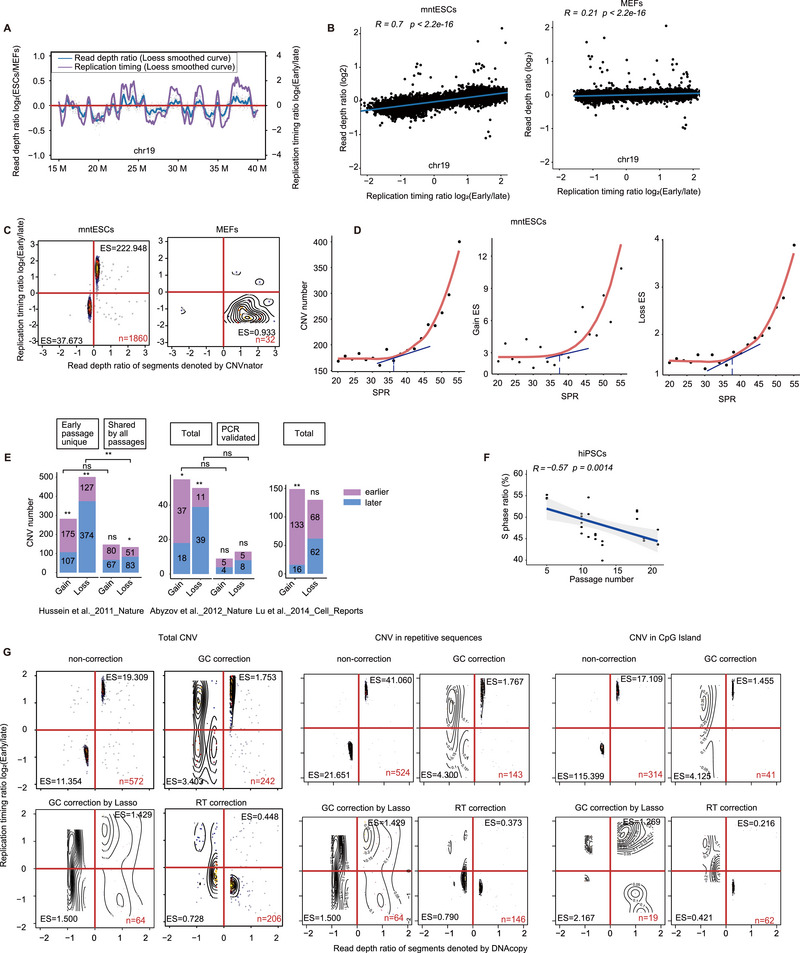
Pseudo‐CNVs exist along high SPR. A) Log_2_ ratio of RD and RT in mntESCs and MEFs at chromosome 19. RD: read depth. B) Correlation between RD and RT ratio in mntESCs (left) and MEFs (right) of chromosome 19. Pearson's correlation coefficient *R* represents the linear correlation between two variables. The *p*‐value was obtained using a *t*‐test. A *p*‐value less than 0.05 was considered indicative of statistical significance. C) Scatter plot of CNV segments determined from the RD and RT profiles of mntESCs (left) and MEFs (right). Most gain or loss segments (ES = 222.948 for gain; ES = 37.673 for loss) in mntESCs were located in early or late RT, respectively. ES: enrichment score. *n*: number of CNV segments. D) In mntESCs, simulation results show that SPR correlates with CNV number (left), ES of CNV gain in early RT (middle), and ES of CNV loss in late RT (right). E) Bar graphs showing CNV distribution in RT from the datasets of Hussein et al., 2011 (left), Abyzov et al., 2012 (middle), and Lu et al. (2014) (right). An imbalanced distribution of CNVs was observed in early passage or during PCR validation. Statistical significance was assessed using Fisher's or χ2 test for comparisons between approaches, and a binomial test for comparisons within groups. ns: not significant and *p* > 0.05; *p < 0.05; **p < 0.01. F) Negative correlation between the SPR and passage number. The Pearson correlation coefficient *R* was −0.57. The *p*‐value was obtained using a *t*‐test. A *p*‐value less than 0.05 was considered indicative of statistical significance. G) Scatter for CNV segments determined from the RD and RT profiles in mntESCs in total (left), repetitive sequences (middle), and CpG Islands (right). In each subplot, the ES of CNV in RT is displayed separately, non‐correction (top left), corrected GC content (top right), corrected GC content by Lasso (bottom left), and corrected RT (bottom right). *n*: number of CNV.

Mouse nuclear‐transferred ESCs (mntESCs) and mouse embryonic fibroblasts (MEFs) exhibit disparate cell cycle phase compositions that reflect distinct differentiation stages.^[^ ^3^ ^]^ In comparison to the MEFs, mouse ESCs (mESCs) demonstrate a diminished G1 duration and a shortened cell cycle phase, with temporal lengths of 12/24 h in MEFs and 3/12 h in mESCs, respectively.^[^ ^33^ ^]^ In this study, we constructed read‐depth profiles of the two cell types to assess the impact of divergent cell cycle phase compositions on CNV identification. MntESCs with a higher SPR exhibited augmented fluctuations in profiles in contrast to MEFs, which had a relatively lower SPR (**Figure** **2**A; Figure S1A). The read‐depth profile of mntESCs exhibited a significant correlation (*r* = 0.7) with the corresponding RTD, whereas that of MEFs did not (*r* = 0.21) (Figure 2B; Figure S1B), suggesting that fluctuations in the read‐depth profile of mntESCs were induced by a higher proportion of cells undergoing replication. The read depth profile was employed to detect CNVs utilizing CNVnator.^[^ ^34^, ^35^, ^36^ ^]^ We discovered that copy number gains were predominantly concentrated in the early replicating domains and, conversely, copy number losses (Figure 2C). Nevertheless, the biased distribution of gains and losses in relation to RTD was not evident in the MEF whole genome sequencing (WGS) data. These findings indicate that elevated SPR elicits false positive signals in CNV identification due to asynchronous DNA replication within the cell population of proliferating cells, such as mntESCs.

Considering the impact of SPR on CNV detection and its dynamic nature during differentiation, we evaluated the extent to which varying proportions of SPR within a cell population would generate significant noise under standard CNV detection parameters. Consequently, a simulation involving a mixture of diverse percentages of S‐phase cells within a cell population was performed. The cell cycle profile is influenced by laboratory and experimental reagents, among other factors.^[^ ^37^ ^]^ Initially, we posited that the effective proportion of SPR cells was 55% in mESCs and 20% in MEFs, based on prior studies.^[^ ^3^, ^38^, ^39^, ^40^ ^]^ Distinct quantities of sequencing reads were sampled and mixed to construct datasets with varying SPR. Subsequently, using CNVnator as the CNV detection software and adhering to a cutoff parameter of 0.25, we discovered that an SPR exceeding 38% in the cell cycle composition precipitated a sharp escalation of pseudo‐CNVs induced by DNA replication (Figure 2D). We also examined the influence of distinct SPRs on CNV detection across various sequencing depths using simulated datasets. Consistent with our expectations, SPRs produced a greater number of false‐positive signals at low sequencing depths (Figure S1C). For ESCs and highly proliferative neoplastic cells, SPRs ranging from 42% to 70% (exceeding 38%)^[^ ^3^, ^41^ ^]^ indicated the presence of false‐positive CNVs within these samples, particularly for low‐coverage WGS data.

To evaluate our hypothesis, we procured CNV calling outcomes of ESCs based on read depth across three published investigations. Intriguingly, the gains and losses were allocated inequitably between the early and late replication domains in all three studies. CNV gains were prevalent in the early replication domains, whereas CNV losses were inversely distributed (Figure 2E). Comparatively, a disproportionate allocation of CNV was not detected in late‐passage human induced pluripotent stem cells (hiPSCs) or in PCR‐validated results that traversed CNV breakpoints (Figure 2E). Selection could explain the diminishing number of CNVs throughout prolonged passage but was not applicable to the unbalanced distribution of augmentations and deficiencies in early passage hiPSCs.^[^ ^42^ ^]^ Furthermore, we cultured ESCs and observed that the SPR progressively declined during the passage of ESCs; by the 20th generation, the SPR diminished to ≈45%, which is close to the tangential point delineated in our preceding simulation (Figure 2D,F; Tables S2 and S3), and an elevated SPR may cause excessive and unevenly distributed pseudo CNVs in early passage hiPSCs. This extrapolation was supported by breakpoint‐checking PCR experiments that reflected the indiscriminate distribution of validated CNV and a relatively low validation rate of CNV.^[^ ^36^ ^]^ PCR results predicated on breakpoint verification can circumvent the influence of read depth and are more accurate than methodologies that are solely dependent on read depth. In summary, we deduced that high SPR in rapidly proliferating cells, such as ESCs, hiPSCs, and cancer cells, would elicit false‐positive CNV due to asynchronous replication in an extended‐S phase cell cycle composition.

Given that SPR affects CNV identification based on read‐depth profiling of a highly proliferative cell population, we intend to rectify this interference to enhance the precision of mutation detection. Prior investigations have demonstrated that GC content variation in mammalian genomes exhibits a robust correlation with the temporal dynamics of replication,^[^ ^43^ ^]^ and GC content has been extensively utilized to mitigate noise in CNV identification based on read depth.^[^ ^18^ ^]^ We subsequently assessed whether GC‐content normalization could alleviate the noise introduced by RT by comparing GC‐content‐normalized data with untreated and RTD‐corrected data. Notably, even after adjusting GC content, the read depth profile manifested elevated noise and RT correlation, in contrast to the RTD correction (Figure S1D,E). This outcome indicates a deficiency in GC content in the RT correction. Furthermore, we evaluated CNV enrichment in repetitive sequences and regions with high GC content (CpG Islands). The findings revealed that the CNV identified following RT correction did not exhibit a pronounced trend of early gain and late RT loss, in comparison with those identified following GC correction or non‐correction (Figure S1D,E). Correspondingly, RTD correction was superior in terms of noise attenuation and could rectify the imbalanced distribution of CNV in accordance with the early and late RTD (Figure 2G). In conclusion, we posited that for an elevated SPR cell sample, RTD correction is imperative prior to CNV identification based on the read depth profile. In particular, for samples with an SPR exceeding 38%, the false‐positive influence of RTD should be considerable in CNV detection.

### 2.2 Chromatin Accessibility was Disrupted by Distinct Cell Cycle Phase Compositions

**Figure 3:**
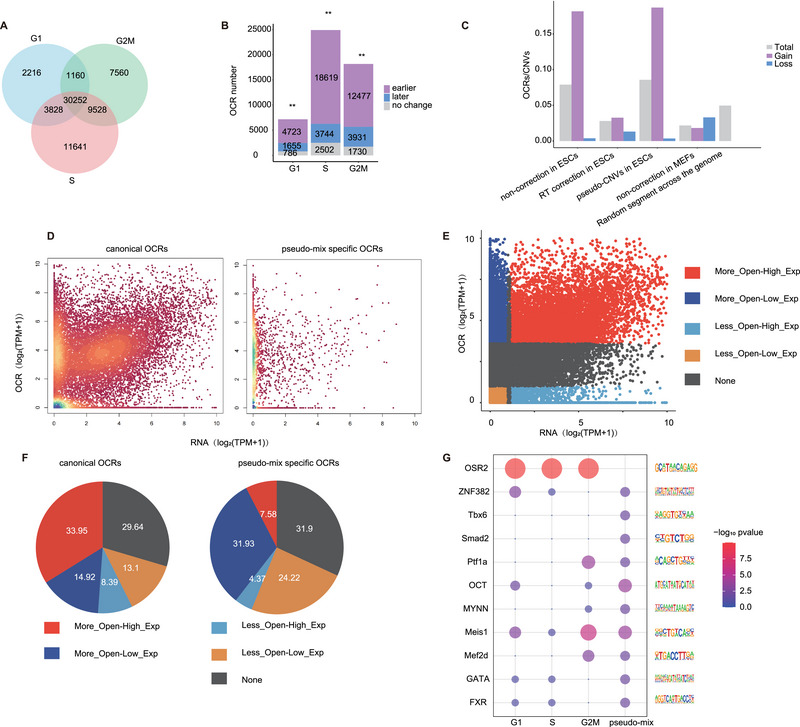
High SPR cells produced false‐positive signals of OCRs. A) Venn diagram showing the OCR distribution in each cell cycle phase. B) The stacked plot shows the number of specific OCRs in each phase with RT. The *p*‐value was calculated using the binomial test. ns: not significant and *p* > 0.05; *p < 0.05; **p < 0.01. C) The bar graphs describe the ratios of OCRs within CNVs in mESCs and MEFs; false‐positive CNVs have a higher ratio than others, and OCRs exhibited a higher enrichment within gain regions compared to loss regions. D) The plot shows the distribution of genes at RNA and OCR in canonical OCR (left) and specific OCR in pseudo‐mix (right). E) The scatter plot shows the distribution in five groups. More_Open‐Hi_Exp: more open and high expression (*n =* 6867), More_Open‐Lo_Exp: More open and low expression (*n =* 7126), Less_Open‐Hi_Exp: less open and high expression (*n =* 3318), Less_Open‐Lo_Exp: less open and low expression (*n =* 22 189), and None (*n =* 17 125). F) The pie shows the proportion of canonical OCRs in the cell cycle phase and specific OCRs in the pseudo‐mix of five groups. G) Dot plot for TFBS in each cell cycle phase and pseudo‐mix data.

Motivated by the influence of the cell cycle on CNV detection, we propose that chromatin accessibility indicators are profoundly related to DNA dosage. Whether OCRs, which are indicative of chromatin accessibility, are influenced by the cell cycle is debatable. Based on previously published evidence that OCRs are susceptible to loci during early RT,^[^ ^22^, ^44^ ^]^ we hypothesized that asynchronous replication would facilitate the unequal distribution of OCRs in relation to RT. Employing the G1, S, and G2M phase chromatin accessibility sequencing data of HCT116 cells, we observed an extensive presence of phase‐specific OCRs (**Figure** **3**A). Furthermore, the chromatin accessibility profile of the S phase was more similar to that of the G2M phase than to that of the G1 phase (Figure S2A). As anticipated, in the assessment results concerning the distribution of phase‐specific OCRs on the RTD, S phase‐specific OCRs were enriched in the early RTD compared to those in G1 and G2M (Figure 3B). To further support the hypothesis that phase‐specific OCRs are induced by asynchronous replication during S phase, we conducted a reanalysis of WGS and ATAC‐seq data for mESCs and MEFs, subsequently integrating the identified OCRs with CNV results across various conditions. Our analysis revealed that OCRs exhibited a significant enrichment within gain regions as opposed to loss regions, particularly in relation to the pseudo‐CNV signals; however, the uneven distribution of OCRs was rectified in the CNVs identified following RTD correction, whereas a similar imbalance in OCR distribution was not evident in MEFs. Furthermore, regarding the high‐confidence CNVs, we do not observe the enrichment of OCRs, by comparing them with the baseline distribution of OCR throughout the genome (Figure 3C). These observations suggest the presence of false‐positive signals of OCRs attributable to the asynchronous replication occurring in S‐phase cells.

We mixed G1, S, and G2M sequencing data with the proportion delineated by the cell cycle phase ratio of HCT116 cells, as a pseudo‐mix sequencing dataset to emulate conventional ATAC data devoid of cell cycle partitioning. We found that the distribution of OCRs at the RTD for the mixed data was between that for the S phase data and that for the G1 or G2M phases (Figure S2B,C). Given that OCRs perform a regulatory function by modulating the expression of adjacent genes, computation of the correlation between OCR signals and the expression levels of OCR‐flanking genes facilitated our assessment of the false positives of OCR signals engendered by DNA replication. The OCRs identified across all cell cycle phases were designated as “canonical OCRs” (the intersection in three cell cycle stages), which were regarded as authentic OCRs, while those excluded from the canonical category were pseudo‐mix specific OCRs, representing the signals introduced by DNA replication. Consistent with expectations, canonical OCRs were correlated with the expression of flanking genes, whereas pseudo‐mix‐specific OCRs were virtually uncorrelated with expression (Figure 3D).

Moreover, we delineated the OCRs into five categories based on the relative chromatin accessibility signals and expression levels of adjacent genes.^[^ ^45^ ^]^ Canonical OCRs were augmented in the more accessible and high‐expression categories, whereas pseudo‐mix‐specific OCRs were predominantly allocated to the more accessible yet low‐expression categories (Figure 3E,F). Gene enrichment analysis revealed that OCRs within the more open chromatin and high expression categories possessed the highest likelihood of being associated with transcription factors. Conversely, OCRs were attributed to the three remaining categories that were predominantly classified as signaling receptor binding, chaperone binding, and MHC protein complex binding, which are less pertinent to the biological characteristics of ESCs (Figure S2D). Further motif analysis indicated that pluripotent transcription factors such as OSR2^[^ ^46^ ^]^ significantly interacted with canonical OCRs; however, this phenomenon was not detected in the pseudo‐mix data (Figure 3G). Therefore, we deduced that the chromatin accessibility profile was susceptible to the cell cycle, such that for cells exhibiting high SPR, cell cycle division, and phase comparison are alternative resolutions.

### 2.3 Methylome Disruption Attributable to Methylation Delay in High SPR Cells

**Figure 4:**
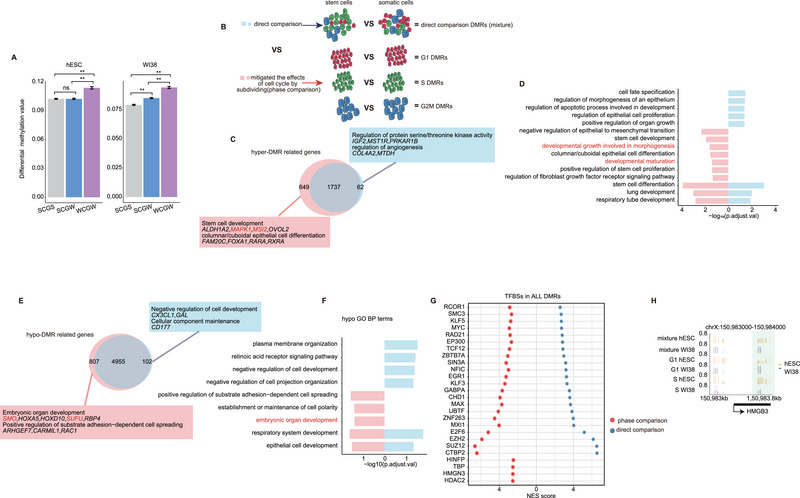
DNA methylation profiles of hESCs and WI38 cells with and without distinction of the cell cycle. A) Difference in the methylation values between the G1 and S phases of the three motifs in hESCs (left) and WI38 cells (right). The WCGW model demonstrated a higher difference value than the other models. The *p*‐value was obtained using the Student's *t*‐test. ns: not significant; *p* > 0.05; *p < 0.05; **p < 0.01. The error bar indicates the standard error (SE). B) The schematic overview showing the influence of cell cycle composition. C) and E) Venn diagram comparing two differential DNA hypermethylation (C) and hypomethylation (E) analyses. The rectangle on the bottom left shows the special GO terms for unique genes under phase comparison, and the rectangle at the top right shows the special GO terms for unique genes under direct comparison. Terms in the phase comparison were more development‐related, whereas terms in the direct comparison were more DNA replication‐related. D) and F) DMR‐related genes were subjected to GO BP terms analysis. Cell cycle‐related and cell development‐related GO BP terms in genes associated with hyper‐DMR (D) or hypo‐DMR (F) were selected for presentation. G) Predicted TFBS based on DMR. H) DNA methylation level in HMGB3. The Y‐axis represents the DNA methylation value, and the X‐axis represents the region. DNA methylation varies among distinct cell cycle phases or a mixture of cell cycle populations.

Previous studies reported contradictory findings regarding the dynamics of DNA methylation throughout the cell cycle. Distinct DNA methylation levels between the S and G1 phases have been observed in HeLa cells,^[^ ^47^ ^]^ whereas several additional studies have indicated that there are no substantial DNA methylation variations among G0, G1, and G2M cells.^[^ ^48^, ^49^ ^]^ Recent studies employing high‐resolution Repli‐bisulfite‐seq and Hammer‐seq have supported a pronounced delay in nascent strand DNA methylation.^[^ ^6^, ^27^ ^]^ To ascertain whether the methylation delay could be discerned through general bisulfite sequencing, particularly in S‐phase cells, we computed the disparity in methylation levels between G1 and S phases across all CpG sites grouped by a 4‐base context (WCGW/SCGW/SCGS).^[^ ^6^ ^]^ We found that actual methylation was delayed in the replication‐uncoupled maintenance regions (**Figure** **4**A, Table S4). Based on this observation, we speculated that delayed methylation of nascent strand DNA would engender pseudo‐methylation disparity signals in a direct comparison between two cell populations with divergent SPRs. Subsequently, we employed methylation data from normal human lung fibroblasts (WI38) and human ESCs (hESCs) across each phase, by comparing the data of unsorted cells, and subsequently assessed whether different cell cycle compositions could influence the detection of epigenetic alterations during state transitions from stemness to differentiation (Figure 4B). A total of 649 specific hyper‐differential methylation regions (DMRs) and 807 hypo‐DMRs were identified in the methylation data between WI38 and hESCs through comparative analysis of each phase, which were not observable in direct comparison without phase discrimination. In addition, 62 hyper‐DMRs and 102 hypo‐DMRs were predominantly identified in the direct comparison (Figure 4C–E; Figure S3A,B). Within the category of genes adjacent to DMRs, genes related to stemness, such as *MAPK1* and *MSI2*,^[^ ^50^ ^]^ were differentially methylated in the cells at specific phases. Conversely, *IGF2* was relevant to the cell cycle solely in the direct comparison (Figure 4C).^[^ ^51^ ^]^ Moreover, for replication‐uncoupled maintenance regions characterized by WCGW (W = A or T) content, referred to as delayed methylation, the DMRs delineated by direct comparison exhibited a higher overlapping ratio^[^ ^6^ ^]^ (Figure S3C). These observations confirm that false‐positive differential methylation signals arise from a direct comparison between the cells with distinct SPRs.

Additionally, a comprehensive functional analysis was conducted to ascertain whether the DMRs delineated using the aforementioned strategy could accurately reflect the biological disparities between WI38 cells and hESCs. Gene Ontology (GO) analysis of genes adjacent to the identified DMRs, compared with cells across various phases, revealed functional terms pertinent to differentiation, including “developmental maturation”, “developmental growth involved in morphogenesis”, and “embryonic organ development” (Figure 4D–F; Figure S3D,E). The transcription factor binding site (TFBS) analysis of the DMRs indicated that, in contrast to direct comparison, the analysis with cells across different phases yielded more robust DMR signals that were significantly associated with transcription factors crucial for lung fibroblast development, such as *HINFP*, *TBP*,^[^ ^52^ ^]^ *HMGN3*, and *HDAC2* ^[^ ^53^ ^]^ (Figure 4G). *HINFP* is a pivotal regulator of lung development.^[^ ^54^ ^]^ In comparison, the methylation levels of the *HMGB3*‐targeting *HINFP* gene increased in G1 phase cells, but this was not observed in S phase or unsorted cells (Figure 4H). Furthermore, genes associated with stemness and differentiation, including *GPR50* (*TBP* targeting),^[^ ^55^ ^]^ *FZD10* (*HMGN3* targeting),^[^ ^56^ ^]^ *PARP10* (*HMGN3* targeting),^[^ ^57^ ^]^ and *FAM215A* (*HDAC2* targeting),^[^ ^58^ ^]^ demonstrated differential methylation signals exclusively in cells at specific phases, which were absent in the mixed cell population (Figure S3F). Collectively, our analysis revealed that methylation delays could induce pseudo‐signals in cells with high SPR, particularly at WCGW sites. Thus, comparing cells within the same phase emerged as the optimal strategy for defining DMRs in samples characterized by high SPR.

### 2.4 Transcriptome Interpretation In Populations with Varied Cell‐Cycle Compositions

**Figure 5:**
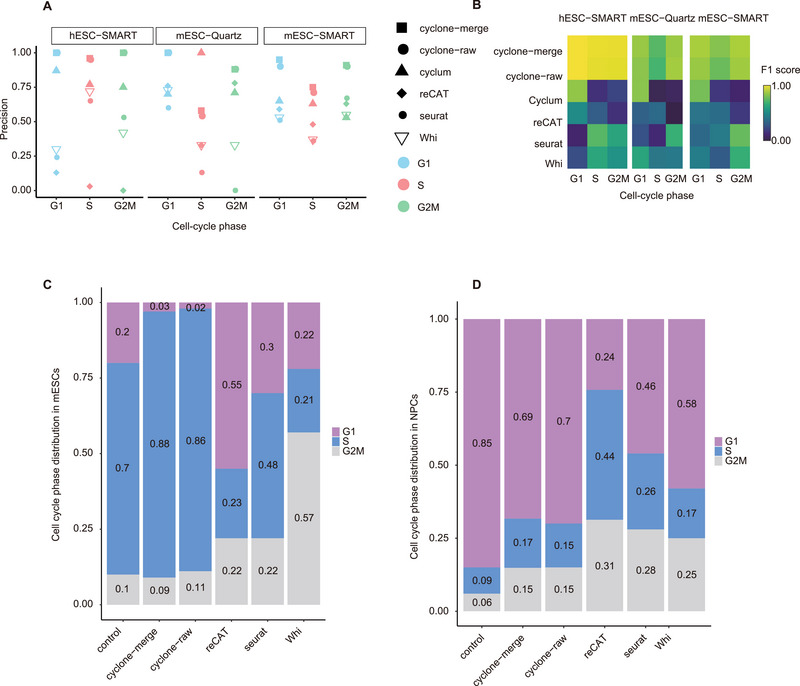
The performance of classifying the cell‐cycle phase. A) Bar plot showing precision in the classification methods. Different colors represent the cell cycle phase. The cyclone‐merge showed a better performance. mESC‐SMART: Hoechst 33 342 mouse ESC(Smart‐Seq protocol); mESC‐Quartz: Hoechst 33342‐stained mouse ESC cell (Quartz‐Seq protocol); hESC‐SMART: hESCs (Smart‐Seq protocol). Precision: TP/(TP+FP); TP: true positive; FP: false positive. B) Random simulation and resampling are used to compare the performance of the methods. The cyclone‐merge had a higher F1 score (top). The color bar represents F1 score value. C) and D) Bar plots showing classification results in mESCs (183 cells, C) and NPCs (29 cells, D). The cyclone‐merge showed better classification in mESCs.

In contrast to the genomic characteristics attributable to asynchronous replication and delayed modifications, the impact of the cell cycle on the transcriptome has been investigated using the distinct transcriptomic profiles associated with each cell cycle phase. With the widespread application of scRNA‐seq, cell cycle heterogeneity within the single‐cell transcriptome has been extensively recognized.^[^ ^59^ ^]^ Although numerous methodologies for classifying cell cycle stages have been developed, accurately assigning individual cells to the correct cell cycle phases using scRNA‐seq data continues to present challenges.^[^ ^60^ ^]^ Initially, we assessed the efficacy of several widely used cell cycle phasing methodologies, including cyclone‐merge,^[^ ^16^ ^]^ cyclone‐raw,^[^ ^16^ ^]^ cyclum,^[^ ^61^ ^]^ reCAT,^[^ ^60^ ^]^ Seurat,^[^ ^62^ ^]^ and Whi^[^ ^63^, ^64^ ^]^ (**Figure** **5**, Table S5). Utilizing cell phase assignments derived from fluorescent ubiquitination‐based cell cycle indicators (FUCCI) as the gold standard, cyclone‐merge exhibited the highest precision and the highest F1 score (Figure 5A,B). The cell phase ratios estimated through cyclone‐merge, for instance, mESCs: 2.7% G1, 88% S, and 9.3% G2M cells; and mouse neural progenitor cells (mNPCs): 69% G1, 17% S, and 15% G2M cells (Figure 5C,D, Table S6), demonstrated the highest concordance with previous flow separation reports.^[^ ^65^ ^]^ Based on the evidence, we employed a cyclone‐merge methodology to accurately allocate single cells to their respective cell cycle phases and proceeded with subsequent analyses.

**Figure 6:**
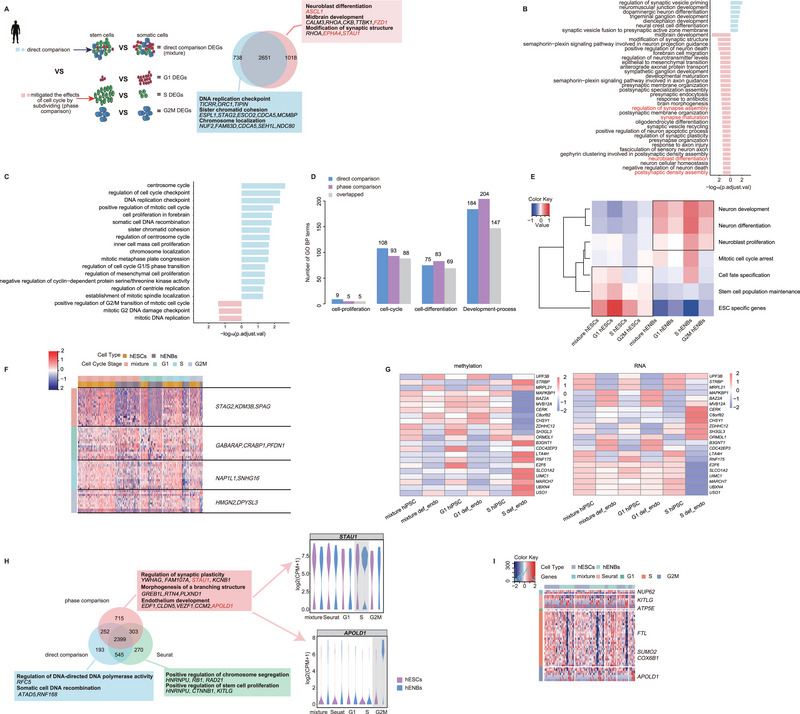
Transcriptome profiles with and without distinguishing between hESCs and hENBs. A) Venn diagram comparing two differential expression analyses in humans. For the left circle, 1018 unique DEGs were identified by phase comparison. For the right circle, 738 unique DEGs were identified by direct comparison. The intersection identified 2651 DEGs shared by both analyses. B) and C) Cell development (B) and cell cycle (C) related GO BP terms were selected for presentation. Phase comparison yielded more development‐related terms and fewer cell cycle‐related terms compared to direct comparison. D) Bar plot showing GO terms for the classification of “cell proliferation”, “cell cycle”, “cell differentiation”, and “development process”. The phase comparison included more cell differentiation‐ and development‐related terms and fewer cell cycle‐related terms than the direct comparison. E) The average expression values of distinct gene signature scores of hESCs and hENBs. The expression of ESC‐specific genes was highest in hESCs of the G1 phase, whereas the expression intensity of genes associated with specific types of cells was highest in hENBs of the S phase or G2M phase. F) Heatmap for selected DEGs. The color bar represents Z‐scores of gene expression values. G) The heatmap shows promoter methylation levels of genes (left) and their corresponding expression patterns (right) in hiPSCs and their differentiation toward definitive endoderm cells. def_endo: definitive endoderm. The color bar represents Z‐scores of gene methylation levels (left) or gene expression values (right). H) Venn diagram comparing three differential expression analyses in human cells. For the bottom left circle, 193 unique DEGs were identified by direct comparison. For the top circle, 715 unique DEGs were identified in the phase comparison, and for the bottom right circle, 270 DEGs were uniquely identified in Seurat. The rectangle shows the special GO terms in each analysis. The terms in the phase comparison were more development‐related terms, whereas the terms in the direct comparison or Seurat were more DNA‐replication‐related terms. I) Heatmap for selected DEGs in the three methods. The color bar represents Z‐scores of gene expression values.

The cell cycle‐partitioned scRNA‐seq data of hESCs (39 G1 phase cells, 99 S phase cells, and 52 G2M phase cells) and human embryonic neuroblast (hENB) cells (75 G1 phase cells, 37 S phase cells, and 15 G2M phase cells) were subsequently employed to assess the impact of the cell cycle on DEGs identification (Figure S4; Tables S6 and S7). We performed a phase comparison, resulting in the final delineation of 2651 DEGs within the intersection outcome. Among these, 1018 DEGs were identified by phase comparison, as opposed to a direct comparison devoid of phasing (**Figure** **6**A; Figure S5A). The predominant portion of the de novo DEGs, including *STAU1* and *EPHA4*, were implicated in neural differentiation and development, thereby reflecting the biological characteristics of hENBs (Figure 6A). GO analysis further supported that DEGs discerned in the comparative analysis for each phase exhibited a superior elucidation of transcriptomic alterations, as they were specifically enriched in the GO terminologies such as “neuroblast differentiation” and “regulation of synapse assembly” (Figure 6B). In contrast, DEGs uniquely identified in direct comparisons, such as *NDC80*, *CDCA5*, and *STAG2*,^[^ ^66^ ^]^ were profoundly associated with DNA replication and enriched in terms pertinent to the cell cycle (Figure 6A,C,D; Figure S5B). Additionally, we constructed a network of specifically enriched GO terms for upregulated DEGs in hENBs. This network illustrated a heightened relevance to neuronal development, as exemplified by “dendritic development”, for the upregulated DEGs in the phase‐based comparative group (Figure S5C,D).

These findings prompted us to investigate the cell cycle phases during which the transcriptome accurately reflected the biological attributes of hESCs and hENBs. Seven distinct gene expression signatures were identified. Three referred to “Neuron development”, “Neuron differentiation”, and “Neuroblast proliferation” reflected the neuron development, while the remaining signatures were “Mitotic cell cycle arrest”, “Cell fate specification”, “Stem cell population maintenance”, “ESC specific genes”, which reflected the cell cycle. Notably, both hESCs and hENBs exhibited disparate signature scores across the corresponding cell cycle phases. Among hESCs, G1 phase cells demonstrated the highest scores for signatures pertinent to pluripotency maintenance, whereas genes associated with neuronal development were markedly expressed during the S and G2M phases (Figure 6E, Table S8). These findings corroborate prior investigations indicating that the genes involved in stemness maintenance of mESCs are predominantly expressed during the G1 phase,^[^ ^33^, ^67^, ^68^ ^]^ whereas cell type‐specific genes tend to be highly regulated during the S and G2M phases.^[^ ^69^ ^]^ Furthermore, a phase‐specific expression pattern was observed in individual genes, such as the neuronal development‐associated genes *GABARAP* ^[^ ^70^ ^]^ and *HMGN2* ^[^ ^71^ ^]^ (Figure 6F). Similar phenomena were observed in the comparison between mESCs, mNPCs, and MEFs (Figures S6 and S7), and phase‐specific gene expression profiles were experimentally validated using RT‐qPCR (Figure S6J).

To further support the influence of DEG identification and the downstream interpretation of the biological relevance, we integrated the methylation and expression data of hiPSCs and differentiated definitive endoderm cells at distinct cell cycle phases. We observed phase‐specific expression patterns in cell cycle‐sorting samples that were not identified in the mixture samples without sorting, and the expression pattern was supported by the methylation levels of the promoter regions for the corresponding genes (Figure 6G). These results reiterate that varying phase distributions in stem and somatic cell populations influence the identification of DEGs and the subsequent comprehensive interpretation of biological signals of interest.

In addition to cell phasing, several methodologies have been documented to eliminate cell‐cycle effects from scRNA‐seq data. Here, we compared the performance of single‐cell phasing for the rectification of the cell cycle effect with the Seurat approach, utilizing the scRNA‐seq data of hESCs and hENBs. We found that the DEGs identified through phase comparison yielded a greater abundance of informative development‐associated genes in hESCs and hENBs, whereas inadequate rectification of cell cycle influence via Seurat resulted in erroneous interpretations and omission of DEGs (Figure 6H,I). For instance, cell cycle‐associated DEGs, such as *NUP62* and *KITLG*, were identified by Seurat, whereas neuron development‐associated DEGs, such as *FTL* and *APOLD1*, were overlooked in the Seurat methodology. Overall, the comparative strategy for each phase was effective in detecting DEGs that accurately reflected the transcriptomic alterations during development. Furthermore, based on the aforementioned comparative analysis, we established a procedural framework (https://github.com/nryisu/pseudo_omic_features_analysis_pipeline/blob/main/Figure4/deg_preprocess.sh) by integrating the cell cycle phasing and DEG detection processes for scRNA‐seq data, which could delineate the precise DEGs of individual cell clusters after eliminating the influence of distinct cell cycle phase compositions.

## 3 Discussion

In this study, we systematically evaluated the influence of the cell cycle on omics data across various cell types, including DNA copy numbers, chromatin configurations, methylation patterns, and transcriptomic profiles. By employing a series of methodologies, including validated data curation substantiated by diverse biological evidence, pairwise comparative analysis, simulation assessments, biological relevance evaluations, and the integration of multi‐omic datasets, we elucidated the pervasive existence of pseudo‐omic characteristics engendered by extended S‐phase cells. To mitigate the effects of disparate cell‐cycle phase distributions on the interpretation of omics features, we propose a comprehensive set of strategies tailored for the analysis of omics data. For genomic datasets, it is advisable that samples exhibiting an SPR exceeding 38% undergo RTD calibration prior to CNV identification. For other omics data, the division of cell cycles along with subsequent phase comparison proved beneficial for executing pairwise analyses between these samples, which may mitigate the effects of disparities in cell cycle phase compositions on phase comparison among the samples. Ultimately, our approach aimed to enhance the interpretation of omics characteristics within proliferative cells.

Previous investigations reported that elevated SPR within cellular populations would introduce noise pertinent to RTD, thereby compromising the accuracy of CNV identification.^[^ ^4^, ^24^, ^72^ ^]^ In this study, we validated the presence of pseudo‐signals from various aspects. By reassessing the attributes of somatic CNVs discerned between stem and differentiated cell types, we identified a nonuniform distribution of CNVs that exhibited a significant correlation with RTD. The distribution tended to be balanced through extended culture passages and PCR analyses that traversed the breakpoints of CNVs. Furthermore, we established a threshold indicating that ≈38% of SPR could substantially generate pseudo‐CNVs as a consequence of asynchronous DNA replication under standard conditions. Moreover, for these samples, the rectification of the read depth profile in conjunction with the RTD profile prior to CNV calling would be beneficial for mitigating the effects of DNA replication.

The impact of the cell cycle on chromatin accessibility, methylation, and transcriptomics has been observed in previous studies and is ascribed to the unique omics profile characteristic of each phase, commonly referred to as cell cycle heterogeneity.^[^ ^6^, ^8^, ^73^, ^74^, ^75^ ^]^ In this study, we analyzed the variations in chromatin accessibility, methylome, and transcriptome between mammalian stem cells and their differentiated counterparts, subsequently validating the widespread occurrence of false‐positive signals, which ultimately affected the interpretation of the biological significance of these samples. Concerning chromatin accessibility, phase‐specific OCRs, particularly those in the S phase, exhibited an inclination toward earlier RTD and a strong correlation with pseudo‐CNVs engendered by asynchronous DNA replication. These phase‐specific OCRs displayed a reduced correlation with downstream gene expression when compared with canonical OCRs. Cell cycle heterogeneity concerning the methylome is controversial^[^ ^47^, ^48^, ^49^ ^]^; however, our analysis corroborated the existence of methylome heterogeneity across cell cycles by examining the methylation lag in replication‐uncoupled maintenance regions, as delineated by Repli‐bisulfite sequencing data. Notably, DMRs characterized by cells in the concordant phase appeared to accurately reflect the biological attributes of both stem and differentiated cells. With the implementation of scRNA‐seq technology, the heterogeneity of the cell cycle within transcriptomes has been rigorously investigated.^[^ ^76^, ^77^ ^]^ The cell cycle is intrinsically linked to cellular processes and is often considered a confounding variable in DEG detection.^[^ ^78^ ^]^ Our study revealed that variations in cell cycle composition significantly affected the identification of development‐associated DEGs, even after adjusting for cell cycle effects. In contrast, phase comparison proved to be superior in mitigating the impact of the cell cycle and in elucidating authentic transcriptomic characteristics, compared to conventional analytical tools utilized in scRNA‐seq studies. Collectively, our investigation methodically assessed the implications of the cell cycle phase on the interpretation of omics data and offered tailored solutions for various forms of omics data, thereby facilitating a thorough comprehension and management of these data sets.

The compositional dynamics of cell cycle phases exhibit significant variability across distinct cell types during developmental and pathological processes.^[^ ^79^ ^]^ Currently, investigations focused on tumor cells, early embryonic development, and stem cells represent prominent scenarios in biological research, in which functional experiments, such as gene knockout or knockdown, may also exert an influence on cellular proliferation. A common characteristic of these specimens is the pronounced disparity in proliferation rates and the distinct compositions of the cell cycle. While the results derived from the comparative analysis of these specimens, concerning gene expression or epigenetic modifications particularly in highly proliferative tumor or stem cells, especially those associated with tumorigenesis or pluripotency, warrant further exploration to determine whether they are genuinely linked to tumor formation and stem cell maintenance or are merely false positive outcomes resulting from the cell‐cycle phase compositions inherent to the rapid proliferative nature of tumor and stem cells.

Our study has several limitations. First, our analysis incorporated a diverse array of public datasets pertaining to different cell types, which constrains the integrative analysis of multi‐omics data, particularly regarding the integration of single‐cell data, as extremely low detection rates and data sparsity hinder our methodological approach from comprehensively elucidating the impact of cell cycle composition on omics data interpretation. Furthermore, investigators typically refrain from evaluating the composition of the cell cycle within samples. Nevertheless, we propose that, in instances where significant signals pertaining to the cell cycle are evident, it is imperative to assess and rectify the phase composition of the sample prior to the detection of differential signals. Additionally, given that omics characteristics associated with each cell cycle phase vary across different samples,^[^ ^15^, ^80^ ^]^ this inconsistency poses a challenge in developing a streamlined automated protocol for the direct comparison of samples exhibiting divergent cell cycle compositions. However, advanced deep learning methodologies, including causal inference methods and Generative Adversarial Networks (GAN), have demonstrated promise in managing confounding variables,^[^ ^15^, ^81^ ^]^ thereby enhancing the precision of differential result identification. However, the efficacy and applicability of these approaches for cell cycle correction across diverse cell types require further investigation. Ultimately, in the context of CNV analysis, which necessitates the availability of corresponding RTD profiles for specified samples, it is noteworthy that not all cells possess RTD profiles. Despite these limitations, our study enabled the community to comprehend the ramifications of cellular cycle composition in the interpretation of omics data, which could serve as a potential avenue for future investigation.

## 4 Experimental Section

### The Approach for Exploring the Influence of Cell Cycle Composition

To explore the influence of cell cycle composition on omics features, the divergence of stem and differentiated cell populations were analyzed by direct and cell cycle phase comparisons (Figure 1A). Direct comparison was used to compare different cell populations without discriminating the cell cycle phase, and phase comparison was used to distinguish the cell cycle phase, pairwise compare their differences, and merge them. To investigate the impact of cell cycle heterogeneity, an analytical framework was established with the following key steps. First, by leveraging prior knowledge of cell cycle dynamics and proliferation, differences were analyzed across diverse samples. Analytical strategies tailored to different omics data types were applied. In WGS, the RTD correction was implemented when the SPR exceeded 38%. For chromatin accessibility, methylation, and transcriptome data, if the biological effects of the cell cycle were not the primary focus, a phase comparison approach was recommended (Figure 1B). For single‐cell data, cell cycle scoring and regression were employed to classify the cell cycle stages, whereas, for bulk data, cell cycle sorting serves as an effective approach for improving the accuracy of the results.

### Cell Culture

The iPSCs from mouse were cultured on 0.1% gelatin‐coated plates in ES‐FBS culture medium (KnockOutTM DMEM, Thermo Fisher Scientific, 10829018), 15% FBS (Biological Industries, 04‐002‐1A), 0.1 mm nonessential amino acids (Millipore, TMS‐001‐C), 50 U mL^−1^ penicillin‐streptomycin (Invitrogen, 15140122), 0.1 mm 2‐Mercaptoethanol (Millipore) and 1000U mL^−1^ LIF supplement (ESGRO, Millipore), with EmbryoMax Nucleosides (Millipore, ES‐008‐D), and 2 mm GlutaMAX (Thermo Fisher Scientific, 35050061). Undifferentiated hiPSCs (kindly provided by Gao Lab, China) were maintained on Matrigel‐coated plates (Corning, USA) under feeder‐free culture conditions with fresh PGM1 PSC culture medium (Cellapy, China) and subcultured with ethyl‐enediaminetetraacetic acid (EDTA) (Cellapy, China) every three or four days. All cultures were grown at 37 °C in 5% oxygen and 5% CO_2_.

### CNV Calling on WGS Datasets

The CNV datasets of Hussein et al.,^[^ ^42^ ^]^ Abyzov et al.,^[^ ^36^ ^]^ Lu et al.^[^ ^34^ ^]^ and the WGS datasets of mntESCs and MEFs were collected (Table S1). For the WGS dataset, preprocessing steps were performed using TrimGalore v0.6.3 to remove adapter sequences and low‐quality reads.^[^ ^82^ ^]^ Subsequently, the reads were aligned to the mm9 reference genome using BWA v0.7.17, and duplicate reads were removed.^[^ ^83^ ^]^ Then, the unique mapping read count at each window of 10 kb length on the reference genome and the read depth ratio were calculated by dividing the median counts. CNVnator v0.4.1, and DNAcopy v1.58.0 were used to call CNV segments. All parameters were set to default settings. The log_2_ ratio was set to 0.25 in CNVnator and DNAcopy. There were no standardized thresholds for defining CNV in general, and the most commonly used threshold was 0.25.^[^ ^35^, ^84^ ^]^ A CNV length of > 5 kb remained.

### Definition of Earlier and later RTD

The RTD was downloaded from www.ReplicationDomain.org, which was primarily based on Repli‐chiq or Repli‐seq.^[^ ^85^ ^]^ The RT scores for copy number gains and losses in MEFs and mntESCs were calculated. In practice, bedtools v2.28.0 intersect were utilized to identify the overlapping region between the CNV and RTD based on the CNV region.^[^ ^86^ ^]^ The score for each CNV was determined by selecting the maximum value. Scores greater than 0 were classified as “earlier” and those less than 0 were classified as “later”. When analyzing chromatin accessibility sequencing data like the NicE‐seq dataset, the RT scores greater than 0.25 were classified as “earlier”, while differences less than −0.25 were classified as “later” and the absolute values less than 0.25 were classified as “no change”.

### Random Simulation of the SPR in the mntESC Population

First, the total number of reads for mntESCs and MEFs were denoted as RC_esc_ and RC_mef_, and the total read numbers of the S phase in mntESCs and MEFs were calculated. Then, the SPR from 0.52 to 0.30 was recorded as S_ratio_, and its step size was set to 0.02. Next, x reads from the mntESCs and y reads from the MEFs were randomly sampled. X and y must satisfy the following equations:

(1) $$x*RC_{esc}+y*RC_{mef}=RC_{esc}$$

(2) $$x*S_{esc}*RC_{esc}+y*S_{mef}*RC_{mef}=S_{ratio}*RC_{esc}$$

S_esc_ and S_mef_ represent the SPRs of mntESCs and MEFs, respectively. Here, S_esc_ = 0.55, S_mef_ = 0.20, RC_esc_ =  188 502 781, and RC_mef_ =  380 641 607. For example, if S_ratio_ = 0.52, then x =  0.9142857 and y = 0.04244775, according to the equation.

After randomly sampling the reads, the processing of reads was the same as that of the WGS datasets, and CNVs segmentation was called using CNVnator. Finally, the SPR was compared with the number of CNV segments and the enrichment scores of the CNV gain or loss segments. Enrichment score was calculated as follows:

CNV gain enrichment score:

(3) $$\frac{\mathrm{the}\;\mathrm{number}\;\mathrm{of}\;\mathrm{gain}\;\mathrm{in}\;\mathrm{early}/\mathrm{loss}\;\mathrm{in}\;\mathrm{early}}{\mathrm{the}\;\mathrm{total}\;\mathrm{number}\;\mathrm{of}\;\mathrm{gain}/\mathrm{loss}}$$

CNV loss enrichment score:

(4) $$\frac{\mathrm{the}\;\mathrm{number}\;\mathrm{of}\;\mathrm{loss}\;\mathrm{in}\;\mathrm{late}/\mathrm{gain}\;\mathrm{in}\;\mathrm{late}}{\mathrm{the}\;\mathrm{total}\;\mathrm{number}\;\mathrm{of}\;\mathrm{loss}/\mathrm{gain}}$$

To evaluate the effect of sequencing depth on CNV calling, the sequencing depth of the data was assessed using PanDepth v2.5. Then, the DownsampleSam function from Picard v2.1.1 was used to randomly sample reads to sequencing depths of 1 ×, 5 ×, and 10 × for subsequent analysis.

### CNV Calling Via Correction

The GC content correction method used was described by Yoon et al.^[^ ^87^ ^]^ Initially, the read depth was calculated in 10 000 bp windows with a 1‐bp overlap. Next, the GC percentage for each window was applied to correct the read depth, whereas the LASSO method used residuals for correction. The corrected RT method was similar to the GC content‐corrected method.^[^ ^87^ ^]^ First, the read depth in a 10000‐bp window was calculated with a 1‐bp overlap. Subsequently, based on known replication profiles, the RT ratio was normalized by dividing it by the maximum RT value. The read depth with the corrected RT is described below.

(5) $${\tilde{RD}}_{i}=RD_{i}*\frac{m}{m_{RT}}$$

where, ${\tilde{RD}}_{i}$ was the read depth of the *i*th window,  *m_RT_* was the median read depth of all windows with the same normalized RT ratio as that of the ith window, and m was the overall median of all windows.

To explore the potential association between false‐positive CNVs and OCRs, overlapping regions were obtained between OCRs in mESCs and MEFs, and the CNVs derived from uncorrected RT, RT‐corrected CNVs, and false‐positive CNVs. Then the length overlap ratio between the OCRs and CNV regions were calculated. Additionally, 1000 random samplings were performed across the entire genome to generate random regions of the same size as the false‐positive CNV regions, which served as random false‐positive CNV controls, and calculated their average values.

### RNA‐Seq Analysis

For RNA‐seq data, STAR v2.7.1a was used to map the reads to the hg19 reference genome.^[^ ^88^ ^]^ The number of reads on each gene was counted using featureCounts v1.6.4.^[^ ^89^ ^]^

### NicE‐seq Data Analysis

To obtain the OCRs, the data were preprocessed according to a previously described study.^[^ ^10^ ^]^ In brief, TrimGalore v0.6.3 was utilized to remove adapters and low‐quality reads.^[^ ^82^ ^]^ Reads were aligned to the hg19 reference genome using bowtie2 v2.2.3.^[^ ^90^ ^]^ Following the removal of duplicates and mitochondrial reads, OCR was determined using MACS2 v2.1.1.^[^ ^91^ ^]^ If the overlap of the OCR exceeded 1 bp, the regions were combined. The OCRs were fused to the corresponding replication domains for comparison. Next, to make the pseudo‐mix, with G1:S:G2M = 5:3:2,^[^ ^92^, ^93^ ^]^ 100% G1 unique reads, 23.38027% S reads, and 14.59481% G2M reads were randomly sampled. The number of reads on the gene and its upstream 2 kb were also counted, then the transcripts per million (TPM) value was calculated. Phase‐specific OCRs were defined as specific OCRs in each cell cycle phase compared to the pseudo‐mix (the difference between the two). Canonical OCRs were the OCR shared by three cell‐cycle phases, which were not influenced by the cell cycle, and non‐canonical OCRs were specific OCRs in the pseudo‐mix compared to canonical OCRs.

To compare OCRs and their expression, it was classified into five groups as follows^[^ ^45^ ^]^: more open and high expression (More_Open‐Hi_Exp), more open and low expression (More_Open‐Lo_Exp), less open and high expression (Less_Open‐Hi_Exp), less open and low expression (Less_Open‐Lo_Exp), and None. More open (More_Open) genes were defined as the maximum number of read counts for open regions overlapping genes that were higher than the 75th percentile of the data, while less open (Less_Open) genes were defined as having medium‐low open if the TPM was less than one. Similarly, if the TPM of the genes was higher than the 75th percentile of the data, they were defined as highly expressed (Hi_Exp), whereas if the TPM of genes was less than one, they were defined as medium‐low expression genes (Lo_Exp). The sva v3.35.2 package was used to correct the batch effect,^[^ ^93^ ^]^ the batch set “1,1,1,1,2,2” for NicE‐seq and RNA‐seq to construct the design matrix. ClusterProfiler v 3.14.3 was used to perform enrichment analysis,^[^ ^94^ ^]^ and Homer v4.11 was used to predict the TFBS.^[^ ^95^ ^]^

### DNA Methylation Data Analysis

The reads were mapped to the hg38 reference genome using the Bismark v0.22.1.^[^ ^96^ ^]^ DMRs were assessed using the Methylkit v1.10.0 with a cutoff percent methylation difference larger than 30% and false discovery rate (FDR) <0.01 for sensitivity analysis.^[^ ^97^ ^]^ DMR annotation was retrieved using the annotatr v1.10.0 R package.^[^ ^98^ ^]^ DMRs with a methylation difference percentage was >30% were used to predict TFBS using i‐cisTarget, and the cutoff of normalized enrichment scores (NES) were larger than 2.5.^[^ ^99^ ^]^

According to Ming et al., 2020,^[^ ^6^ ^]^ CpG sites can be partitioned into WCGW, SCGW, and SCGS categories, and the solo‐WCGW has a higher DNA methylation delay. Thus, only the solo‐WCGW (0 flanking CpG site at 100 bp) for DNA methylation delay analysis was considered.

The following steps were performed for the integrated analysis of DNA methylation and RNA expression: 1) The single‐cell methylation BAM files for cells in the G1 phase were merged into a G1 group using SAMtools v1.9 merge. Similarly, the BAM files for cells in the S and G2/M phases were merged into an S group, and all cells were incorporated into a mixture group. CpG site methylation information was extracted using the bismark_methylation_extractor in the Bismark tool. 2) The promoter regions were defined as 2 kb upstream to 500 bp downstream of the transcription start site (TSS) based on the reference genome. CpG sites within the promoter regions were quantified using Bedtools, and average methylation levels were calculated. Each gene was required to cover at least 10 CpG sites to ensure data reliability. 3) Differential expression analysis of single‐cell RNA data was performed using the MAST v1.10.0 R package, with |FoldChange| >2 and FDR <0.05. 4) Methylation‐related genes were selected based on specific genes in the direct and phase comparison results, and the mean expression levels of these genes were calculated.

### scRNA‐seq Data Analysis

Three datasets contained cell cycle stage labels sorted by flow cytometry: Hoechst 33342‐stained mouse ESCs (Quartz‐Seq protocol), Hoechst 33 342 mouse ESCs(Smart‐Seq protocol), and FUCCI H1 hESCs(Smart‐Seq protocol). The two unlabeled datasets included mESCs, mNPCs, hESCs, and hENBs. ScRNA‐seq data were processed using the following procedure: First, TrimGalore v0.6.3 was used for quality control. Reads were then mapped to the mm10 or hg38 reference genome using STAR v2.7.1a, and expression values were calculated using featureCounts v1.6.4, based on the GENCODE annotation. Subsequently, low‐expression genes and low‐quality cells were filtered using the scater v1.12.2 R package; for example, removing a high percentage of mitochondrial gene expression cells or reserved genes with at least 5% expression.

Finally, DEGs were retrieved using the MAST v1.10.0 R package with a cutoff of |FoldChange| > 2 and a false discovery rate (FDR) < 0.05.^[^ ^100^ ^]^ Statistical tests were based on the chi‐square test to obtain p‐values, followed by the Benjamini‐Hochberg correction to calculate the FDR. DEGs with a fold‐change value greater than 0 were defined as up‐regulated genes. GO biological process (GO BP) and GO‐Slim term enrichment analyses were performed using ClusterProfiler and GOSemSim v2.10.0 R packages with a Benjamini‐corrected *p*‐value of < 0.05. An enrichment network was constructed using Metascape.^[^ ^101^ ^]^ All parameters were set to their default values. Signature scores were calculated according to Neftel et al.,^[^ ^102^ ^]^ and the gene sets associated with specific biological functions were shown in Table S8.

### Cell Cycle Analyses and Correction

The difference between cyclone‐merge and cyclone‐raw was the gene list used. Cyclone‐merge applied the gene sets that combined the default gene set with the defined gene set from Whitfield et al. (Table S7),^[^ ^64^ ^]^ and cyclone‐raw only used the default gene sets. The average expression profile across cells based on a particular cell cycle phase was calculated as described by Whitfield. The correlation between each gene and the average profile was calculated. Genes with a correlation of <0.1 were excluded (weak). Therefore, these gene sets were combined with the default cyclone gene set to predict the cell cycle phase. Moreover, t‐SNE was used to visualize the cells based on these cell cycle gene sets to determine whether the cells were assigned phase correction.

Cell cycle profiles were determined by fluorescence‐activated cell sorting (FACS) as the ground truth. The accuracy of the cell cycle classification methods was then evaluated by randomly resampling 90% of the cells in the three FUCCI datasets and calculating the following indicators:

(6) $$F1s\mathrm{core}=\frac{2\;*\;\mathrm{sensitivity}\;*\;\mathrm{prescion}}{\left(\mathrm{sensitivity}\;+\;\mathrm{prescion}\right)}$$

(7) $$\mathrm{Sensitivity}=\frac{\mathrm{True}\;\mathrm{Positives}}{\left(\mathrm{True}\;\mathrm{Positives}\;+\;\mathrm{False}\;\mathrm{Negative}\right)}$$

(8) $$\mathrm{Precision}=\frac{\;\mathrm{True}\;\mathrm{Positives}}{\left(\mathrm{True}\;\mathrm{Positives}\;+\;\mathrm{False}\;\mathrm{Positives}\right)}$$

### qPCR validation of DEGs

mESCs were stained with Hoechst 33342 for 30 min at 37 °C, and subsequently sorted using FACS into distinct cell cycle stages (G1, S, and G2/M). Total RNA as extracted from each cell cycle population using Trizol reagent. Reverse transcription was carried out using the HiScript III RT SuperMix for qPCR (+gDNA wiper) (R323, Vazyme, Nanjing, China). The mRNA expression level was analyzed using RT‐qPCR with the TB Green Premix Ex Taq II (Tli RNaseH Plus) (RR820A, Takara bio, China). The primer sequences used for RT‐qPCR are listed in Table S9.

### Statistical Analysis

The statistical significance of correlations was assessed using Student's t‐tests, whereas the differences in the distribution of RTD in omics data were evaluated using a binomial test. The distribution of CNV in RT was especially evaluated using Fisher's exact test or the χ^2^ test, as appropriate. A *p*‐value or FDR of less than 0.05 was considered statistically significant.

## Conflict of Interest

The authors declare no conflict of interest.

## Author Contributions

R.N. and C.Z. contributed equally to this work. C.Z. and J.C. conceptualized and supervised the project. R.N. performed the analysis. C.Z., J.C., R.N., and J.L. performed manuscript writing, review, and editing. Y.T., Y.S., and L.W. provided assistance in writing and analysis. L.R. and R.N. performed cell culture, sorting, and validation experiments.

## Supporting information



Supporting Information

## Data Availability

Data sharing is not applicable to this article as no new data were created or analyzed in this study.
