# Supplementary material for: Mitigating Cell Cycle Effects in Multi‐Omics Data: Solutions and Analytical Frameworks
Source: Adv Sci (Weinh). 2025 May 28;12(29):e05823. doi: 10.1002/advs.202505823 (PMC12362787; doi:10.1002/advs.202505823)
Supplement: Supplementary file 1 — Supporting Information [file ADVS-12-e05823-s001.docx]

**Supplementary**

**Mitigating Cell Cycle Effects in Multi-Omics Data: Solutions and Analytical Frameworks**

Rui Nie^1,2,6^, Caihong Zheng^3,6*^, Likun Ren^1,2^, Yue Teng^1,2^, Yaoyu Sun^4^, Lifei Wang^5^, Junya Li^3^, Jun Cai^1,2*^

^1^ Chinese Academy of Sciences and China National Center for Bioinformation, Beijing 100101, China

^2^University of Chinese Academy of Sciences, Beijing, 100049, China

^3^Laboratory of Molecular Biology and Bovine Breeding, Institute of Animal Science, Chinese Academy of Agricultural Sciences, Beijing 100193, China

^4^School of Life Sciences, Peking University, Beijing 100871, China

^5^Department of Chemistry, the University of Hong Kong, Hong Kong 999077, China

^6^These authors contributed equally: Rui Nie, Caihong Zheng

* Correspondence to: Caihong Zheng, E-mail: zhengcaihong@caas.cn

Jun Cai, E-mail: juncai@big.ac.cn

**
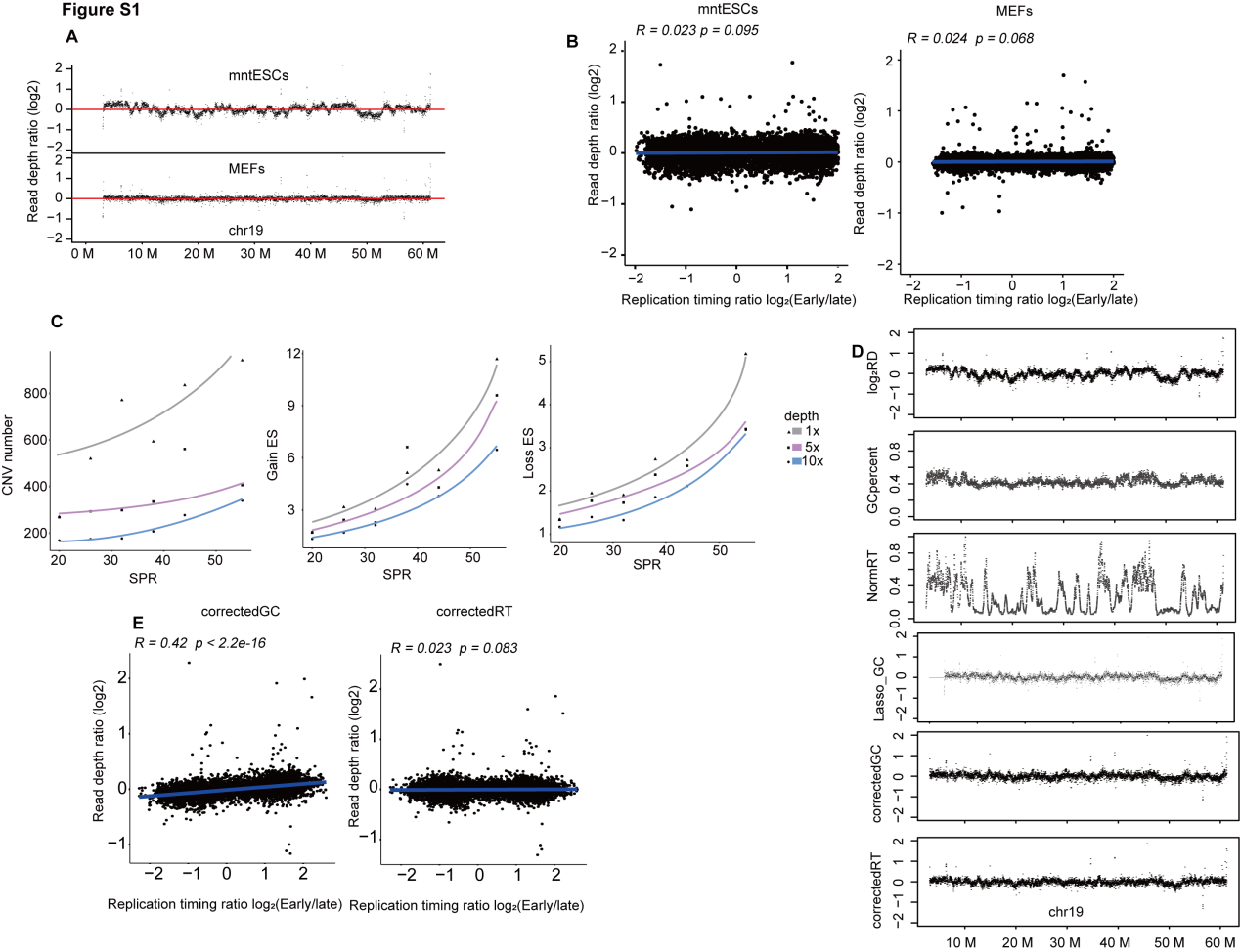
**

**Figure S1. Relationship between RT, GC content, and RD in mntESCs and MEFs.**

**A****)** Scatter plot for normalized RD in mntESCs and MEFs of chromosome 19. **B)** The correlation of random RD and RT ratio in mntESCs (left) and MEFs (right) of chromosome 19. The *p*-value was obtained using a *t*-test. A *p*-value less than 0.05 was considered indicative of statistical significance. **C)** In mntESCs with different read depths, the simulation SPR was related to CNV number (left), ES of CNV gain in early RT (middle), and ES of loss in late RT (right). **D)** The scatter plot for the RD, GC percent, normalized RT, corrected GC content, corrected GC content by Lasso, and corrected RT in mntESCs. **E)** The correlation of corrected GC content (left) or RT (right) in mntESCs of chromosome 19.  The *p*-value was obtained using a *t*-test. A p-value less than 0.05 was considered indicative of statistical significance.


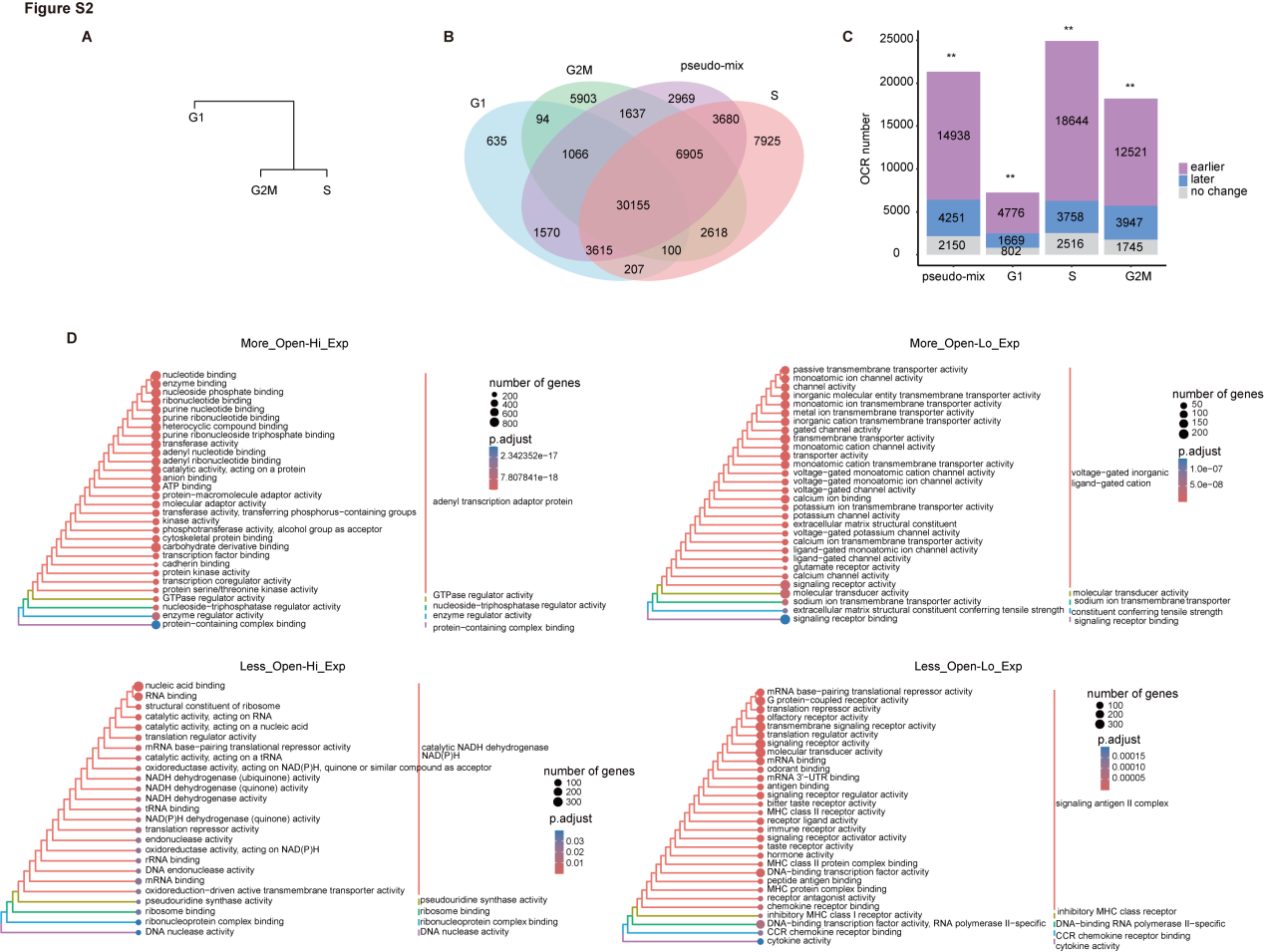


**Figure S2. The distribution of OCRs.**

**A)** Clustering of OCRs in each cell cycle phase based on the TPM value. **B)** The Venn diagram shows the OCR distribution in each cell cycle phase and pseudo-mix. **C)** The stacked plot shows the number of specific OCRs with RT. The *p*-value was calculated using the binomial test. ns: not significant and *p* > 0.05; *: *p* < 0.05; **: *p* < 0.01. **D)** Functional annotation of 4 group-related genes.

**
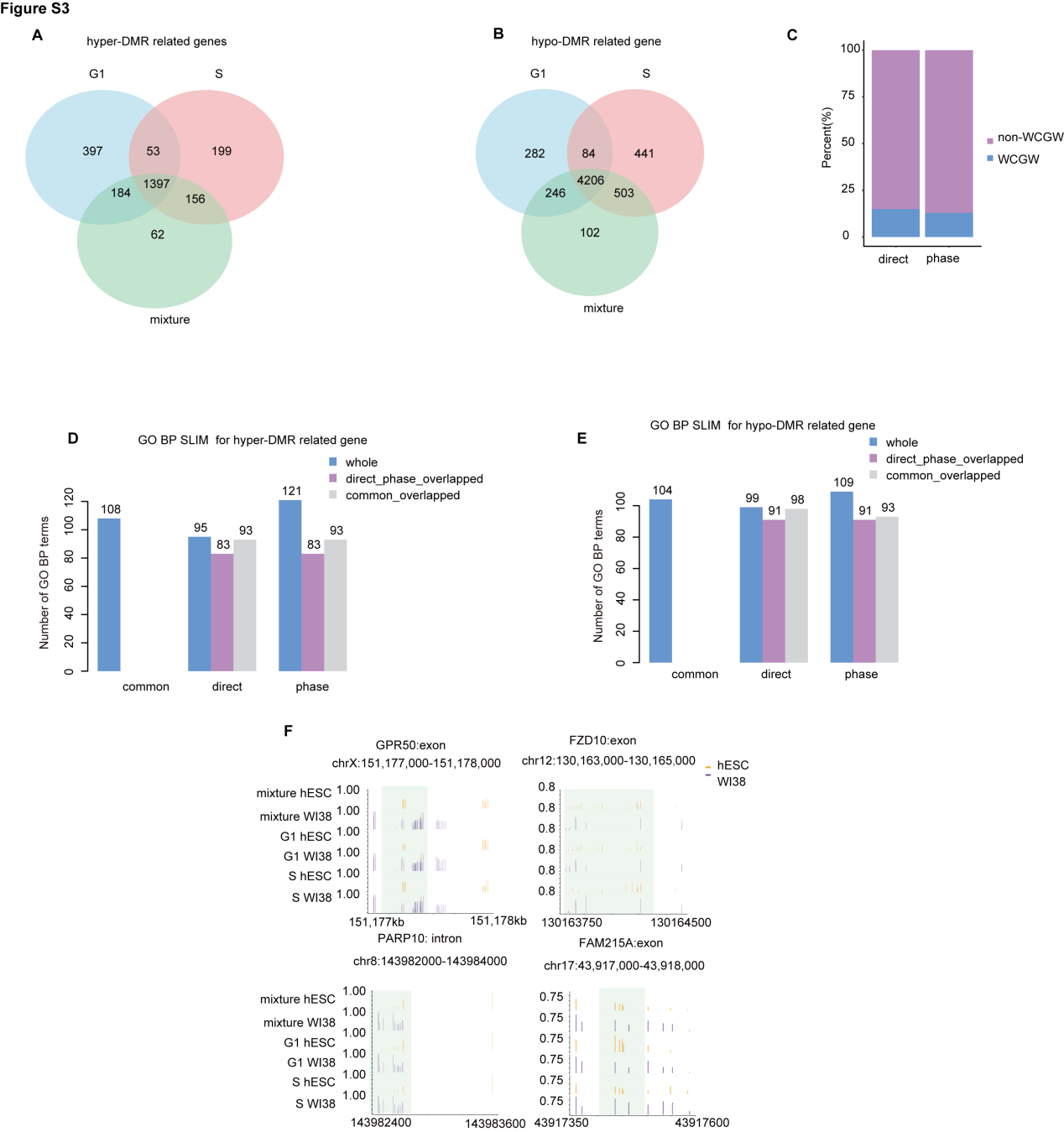
**

**Figure S3. Methylation characterization in humans.**

**A)** and **B)** Venn diagram for mixture, G1, and S differential DNA hypomethylation (**A**) and hypermethylation (**B**) analyses. **C)** The stack plot shows the proportion of DMR-related motif DNA methylation delay (WCGW). WCGW percentage was 15.0% for direct comparison and 12.9% for phase comparison. **D)** and **E)** GO BP Slim term analysis for hyper-DMR (**D**) or hypo-DMR (**E**) related genes. common: same genes in direct and phase comparison for GO BP Slim terms. “whole”: GO BP Slim terms with DMR-related genes in each method; “direct_phase_overlapped”: the overlapped GO BP Slim terms for direct comparison and phase comparison; “common_overlapped”: the overlapped GO BP Slim terms of common and direct or phase comparison. **F)** DNA methylation level in *GPR50* (top left), *FZD10* (top right), *PARP10* (bottom left), and *FAM215A* (bottom right). The Y-axis represents the DNA methylation value, and the X-axis represents the region.


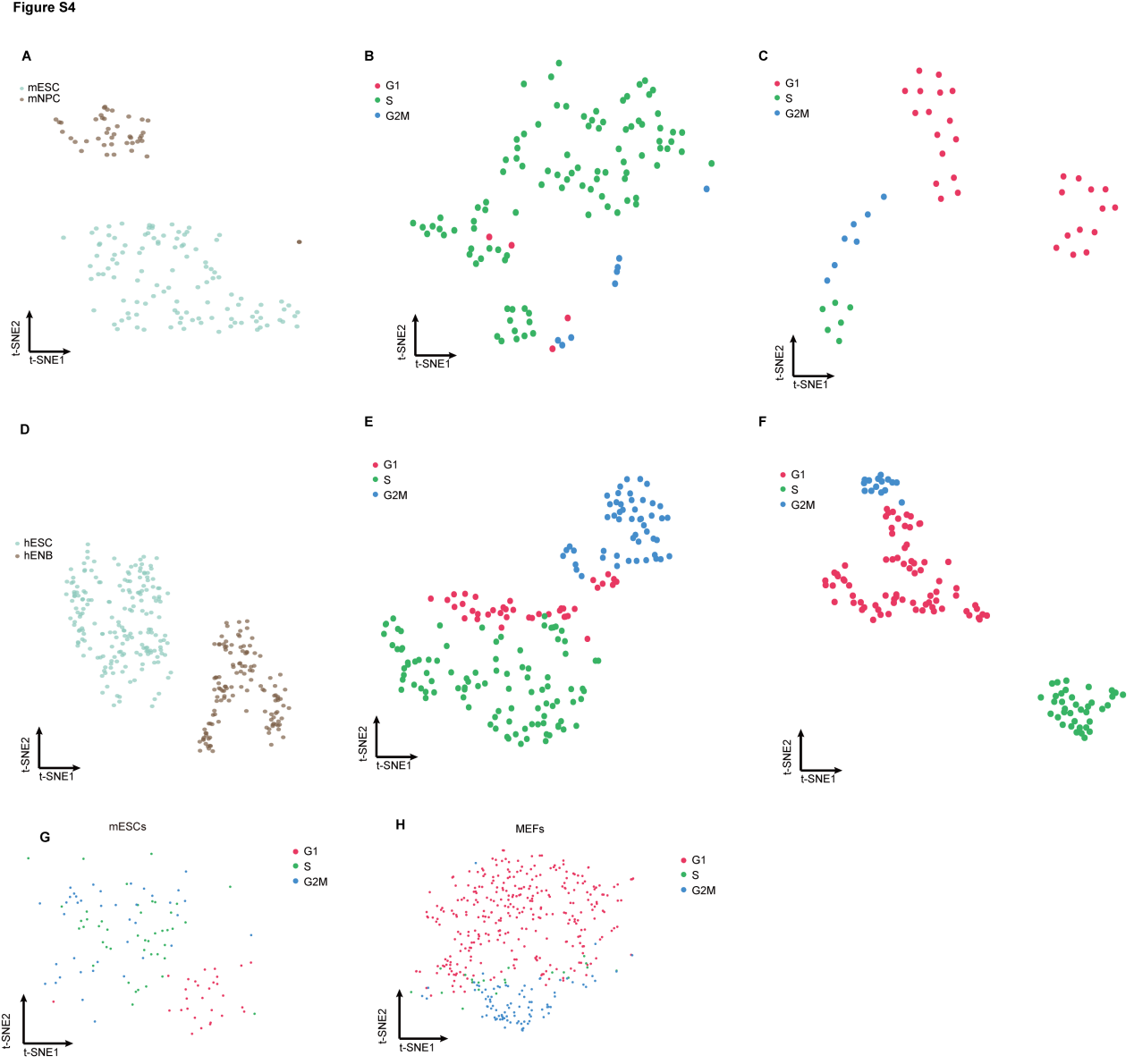


**Figure S4. t-SNE plots of scRNA-seq.**

**A)** mESCs and mNPCs. **B)** mESCs. **C)** mNPCs. **D)** hESCs and hENBs. **E)** hESCs. **F)** hENBs. **G)** mESCs. **H)** MEFs. Individual cells were classified into three cell-cycle stages.

**
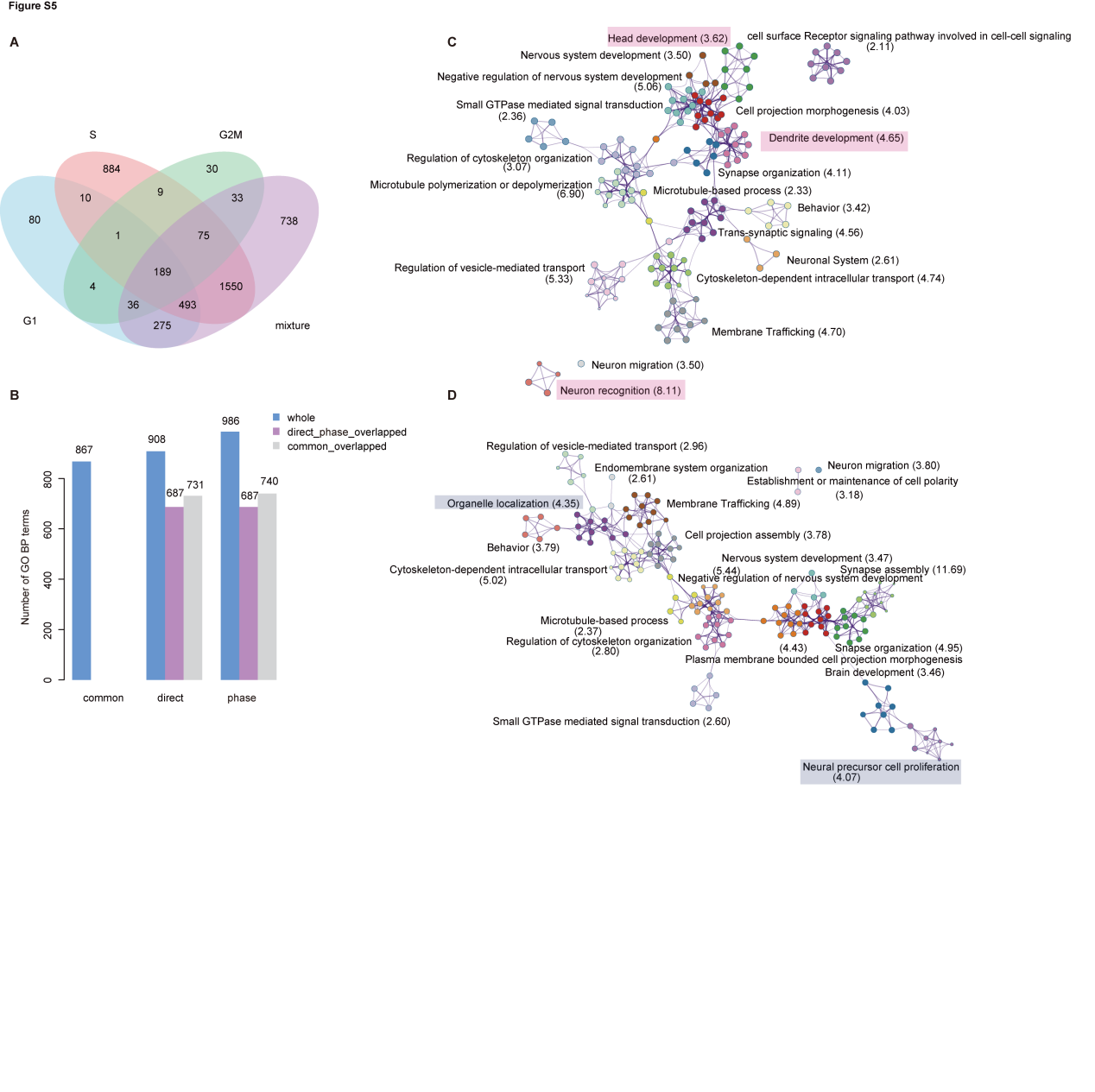
**

**Figure S5. DEGs and functional analysis with or without identification of the cell cycle in humans.**

**A)** Venn diagram for DEGs from mixture, G1, S, and G2M in humans. **B)** The counts for GO BP term analysis for DEGs. common: overlapped genes in direct comparison and phase comparison between each phase for GO BP terms. “whole”: GO BP terms for whole DEGs in each method; “direct_phase_overlapped”: the overlapped GO BP terms for direct comparison and phase comparison; “common_overlapped”: the overlapped GO BP terms of common and direct comparison or phase comparison. **C)**and **D)** Human upregulated DEGs in phase comparison (**C**) and direct comparison (**D**). The gray-shaded part was distinct from the direct comparison, and the phase comparison included more cluster terms related to development.

**
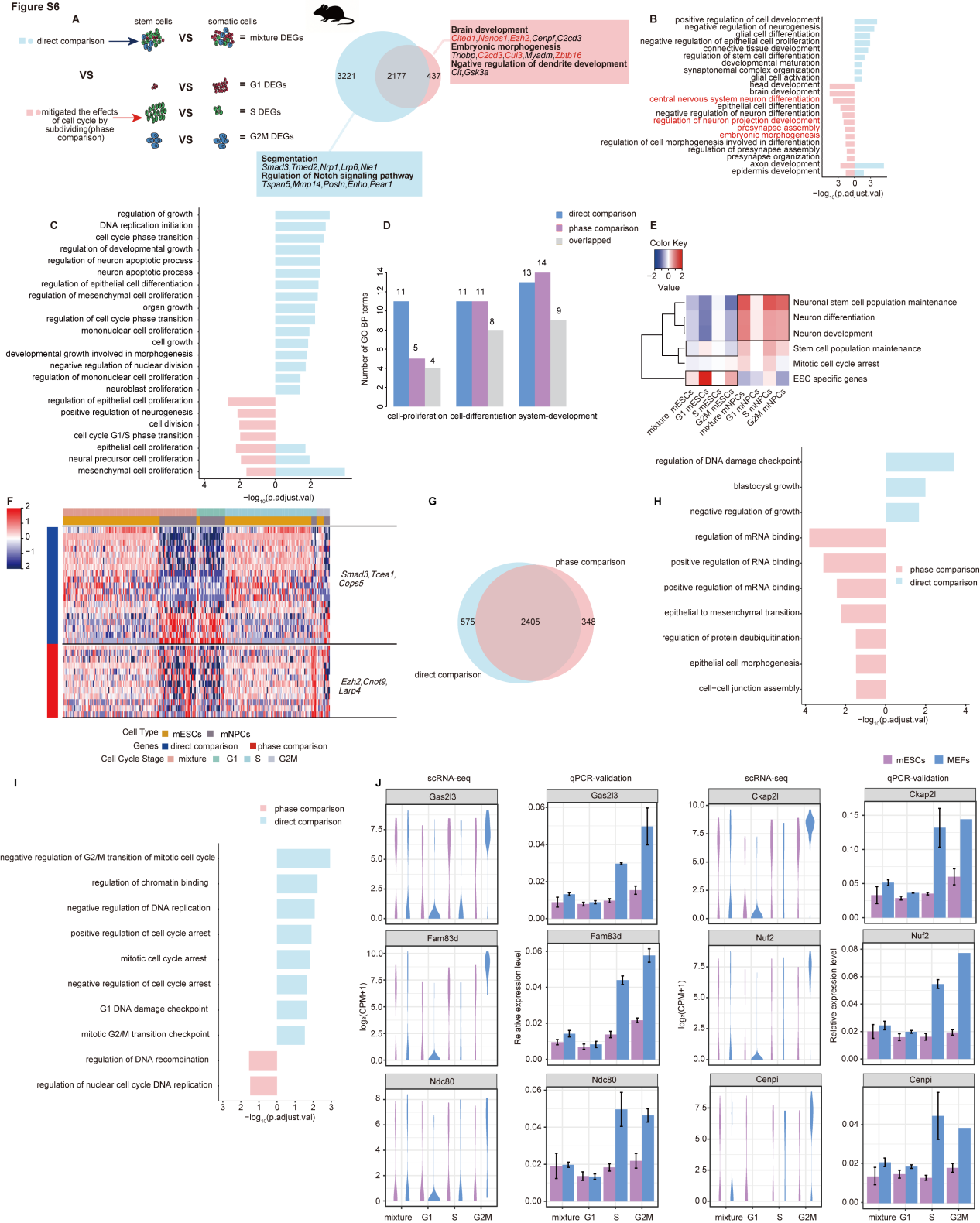
**

**Figure S6. Transcriptome profiles with and without distinguishing cell cycle in mice.**

**A)** Venn diagram comparing two differential expression analyses in mice. For the left circle, 3221 DEGs were uniquely identified by direct comparison of each phase. For the right circle, 437 unique DEGs were identified in the phase comparison. The intersection identified 2177 DEGs shared by direct and phase comparison between mESCs and mNPCs. Cell development (**B**) and cycle (**C**) related GO BP Slim terms were selected for presentation. This shows that phase comparison has more terms than direct comparison in cell development and fewer terms in the cell cycle. **D)** Bar plot shows the counts of GO terms for the classification of “cell proliferation”, “cell differentiation”, and “development process”. **E)** The average expression values of distinct gene signature scores of mESCs and mNPCs. **F)** Heatmap for selected DEGs. The color bar represents Z-scores of gene expression values. **G)** Venn diagram of differentially expressed genes in mESCs and MEFs. **H)** and **I)** Cell development (H) and cell cycle (I) related GO BP terms in mESCs and MEFs were selected for presentation. This shows that phase comparison has more terms than mixture in cell development and fewer terms in the cell cycle. Threshold for differentially expressed genes: abs(Fold change) ≥ 2, FDR < 0.05. **J)** The violin plot showing DEGs identified by phase comparison in scRNA-seq rather than direct comparison mESCs and MEFs. The bar plot showing the qPCR validation results of selected DEGs. Error bars represent standard deviation (SD).

**
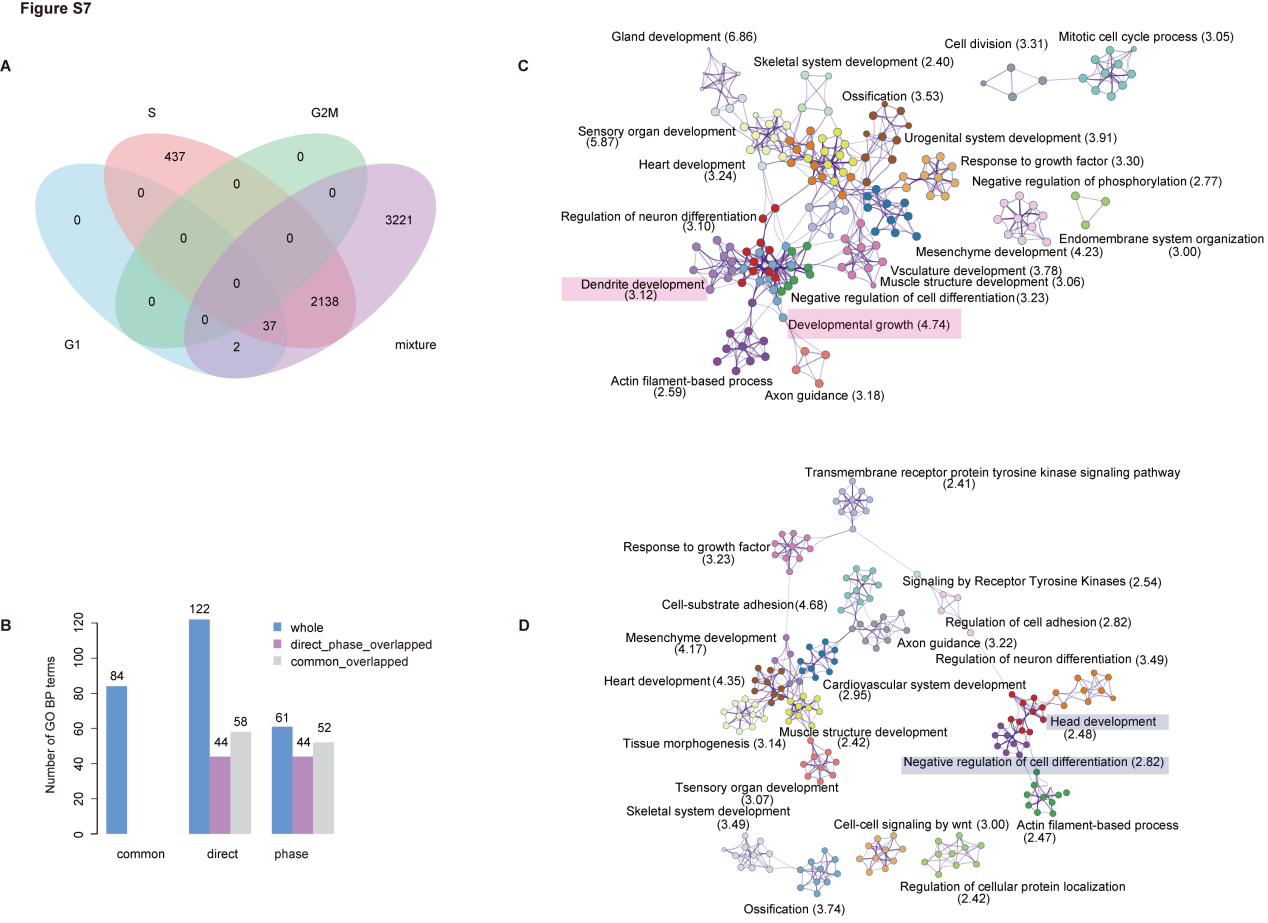
**

**Figure S7. DEGs and functional analysis with or without distinguishing cell cycle in mouse.**

**A)** Venn diagram for DEGs from mixture, G1, S, and G2M in mouse. **B)** GO BP Slim term analysis for DEGs. common: overlapped genes in direct comparison and phase comparison between each phase for GO BP terms. “whole”: GO BP terms for whole DEGs in each method; “direct_phase_overlapped”: the overlapped GO BP terms for direct comparison and phase comparison; “common_overlapped”: the overlapped GO BP terms of common and direct comparison or phase comparison. Mouse upregulated DEGs in phase comparison (**C**) and direct comparison (**D**). The gray-shaded part was distinct from the direct comparison, and the phase comparison included more cluster terms related to development.

Table S1. The available public datasets.

| **Accession** | **Data Type** | **Cell Line** | **PMID** |
| --- | --- | --- | --- |
| NDARSTD289 | DNA-seq | HiPSC lines derived from skin fibroblasts | 23160490 |
| GSE26173 | Affymetrix SNP arrays | HiPSCs from fibroblast lines at early, intermediate and late passage cells | 21368824 |
| GSE46848 | Affymetrix SNP 6.0 arrays | Human fibroblast cells and fibroblast-derived iPS cells | 24685138 |
| DRR029019 | WGS | MEFs | 28233378 |
| DRR029032 |  | Mouse ntESCs |  |
| GSM6067800  GSM6067806 | ATAC-seq | MEFs  mESCs | 35874611 |
| GSM3595431  GSM3595430 | scRNA-seq | mESCs  MEFs | 31067459 |
| ERP016754 | Parallel single cell RNA-Seq and bisulfite sequencing | hiPSC and def_endo | 30744673 |
| SRR10325453 | NicE-seq | G1 phase of HCT116 | 32763232 |
| SRR10325454 |  | G1 phase of HCT116 |  |
| SRR10325455 |  | S phase of HCT116 |  |
| SRR10325456 |  | S phase of HCT116 |  |
| SRR10325457 |  | G2M phase of HCT116 |  |
| GSM1296824 | RRBS | hESC H9 Population rep1 | 25043040 |
| GSM1296825 |  | hESC H9 Population rep2 |  |
| GSM1296848 |  | H9 Cell Line FACS sorted for G1 phase |  |
| GSM1296849 |  | H9 Cell Line FACS sorted for S phase |  |
| GSM1296802 |  | WI-38 Population rep1 |  |
| GSM1296803 |  | WI-38 Population rep2 |  |
| GSM1296844 |  | WI-38 CellCycle G1 Phase rep1 |  |
| GSM1296845 |  | WI-38 CellCycle G1 Phase rep2 |  |
| GSM1296846 |  | WI-38 CellCycle S Phase rep1 |  |
| GSM1296847 |  | WI-38 CellCycle S Phase rep2 |  |
| GSE60749 | scRNA-seq | Mouse ESCs and nestin-positive NPCs | 25471879 |
| GSE76381 |  | H9 ESCs and neuroblasts cells | 27716510 |
| E-MTAB-2805 |  | Hoechst 33342 Mouse ESC | 25599176 |
| GSE42268 |  | Mouse ESCs | 23594475 |
| GSE64016 |  | H1-Fucci ESCs | 26301841 |
| SRR5027802 | bulk RNA-seq | mESC | 28157505 |
| SRR5027803 |  |  |  |
| SRR5027804 |  | mNPC |  |
| SRR5027805 |  |  |  |

Note: The table show the accession for publically available datasets used in this study. scRNA-seq: single cell RNA sequencing; RRBS: reduced representation bisulfite sequencing; FACS: fluorescent activated cell sorting; hiPSC: human induced pluripotent stem cell; ESCs: embryonic stem cells; MEFs: mouse embryonic fibroblasts; ntESCs: nuclear transfer embryonic stem cells; NPCs: neural precursor cells.

Table S2. Cell cycle profile of iPSCs and differentiated cells from human and mouse treated with EdU or DAPI.

| **Cell ID** | **Species** | **Cell Type** | **Percentage (%)** | | | | |
| --- | --- | --- | --- | --- | --- | --- | --- |
|  |  |  | G0/G1 Phase | Early S Phase | Late S Phase | G2/M Phase | Total |
| APC | Mouse | Differentiated cell | 46.2 | 5.86 | 9.06 | 35.5 | 96.62 |
| MEF | Mouse | Differentiated cell | 59.2 | 7.42 | 11.4 | 21.2 | 99.22 |
| S-2-1 | Mouse | iPS cell | 15.1 | 39.1 | 35.4 | 8.27 | 97.87 |
| S-2-14 | Mouse | iPS cell | 17.3 | 36.7 | 35.4 | 9.15 | 98.55 |
| S-2-19 | Mouse | iPS cell | 18.4 | 38.3 | 33.2 | 8.97 | 98.87 |
| 6-2-8 | Mouse | iPS cell | 16.3 | 47.1 | 27.8 | 6.5 | 97.7 |
| 6-2-15 | Mouse | iPS cell | 17.6 | 46.6 | 31 | 3.01 | 98.21 |
| 6-1-16 | Mouse | iPS cell | 25.1 | 26 | 27.5 | 16.8 | 95.4 |
| GM05659 | Human | Differentiated cell | 76.5 | 3.54 | 5.83 | 12.1 | 97.97 |
| GM08399 | Human | Differentiated cell | 79.5 | 5.68 | 2.52 | 9.87 | 97.57 |
| LT-4 | Human | iPS cell | 44.7 | 18.1 | 12.3 | 21.6 | 96.7 |
| LT-16 | Human | iPS cell | 38.9 | 18.2 | 16 | 24.1 | 97.2 |

Table S3. The relationship between culture passage number and S phase ratio in hiPSCs.

| **ID** | **Passage** | **SPR (%)** |
| --- | --- | --- |
| hiPSCs | P5 | 55.2 |
| hiPSCs | P5 | 54.5 |
| hiPSCs | P5 | 55.2 |
| hiPSCs | P5 | 54.3 |
| hiPSCs | P10* | 47.7 |
| hiPSCs | P10* | 48.7 |
| hiPSCs | P10* | 46.2 |
| hiPSCs | P10 | 50.2 |
| hiPSCs | P10 | 50.8 |
| hiPSCs | P10 | 50.8 |
| hiPSCs | P11* | 45.4 |
| hiPSCs | P11* | 50.7 |
| hiPSCs | P11* | 54.6 |
| hiPSCs | P12* | 46.1 |
| hiPSCs | P12* | 44.9 |
| hiPSCs | P12* | 45.8 |
| hiPSCs | P13* | 44.4 |
| hiPSCs | P13* | 39.9 |
| hiPSCs | P13* | 43.5 |
| hiPSCs | P18 | 49.6 |
| hiPSCs | P18 | 51.6 |
| hiPSCs | P18 | 51.3 |
| hiPSCs | P19 | 44.5 |
| hiPSCs | P19 | 45.3 |
| hiPSCs | P19 | 45 |
| hiPSCs | P21 | 43.6 |
| hiPSCs | P21 | 43.7 |
| hiPSCs | P21 | 47.1 |

Table S4. CpG site related motif statistics in humans.

|  | reference Genome | RRBS covered CpG site in hESC | RRBS covered CpG site in wi38 | 0 flanking CpG site at 100bp in hESC | G1>S | 0 flanking CpG site at 100bp in wi38 | G1>S |
| --- | --- | --- | --- | --- | --- | --- | --- |
|  |  |  |  |  | delta(G1-S)  value of 0 flanking CpG site at 100bp in hESC |  | delta(G1-S)  value of 0 flanking CpG site at 100bp in wi38 |
| SCGS | 8232685 | 103949 | 96737 | 25934 | 23649 | 24318 | 22354 |
| WCGS | 13690487 | 75399 | 91032 | 15019 | 13983 | 22232 | 20823 |
| WCGW | 7229261 | 20142 | 28559 | 5198 | 4961 | 8581 | 8226 |
| Total | 29401360 | 199490 | 216328 | 46151 | 42593 | 55131 | 51403 |

Table S5. Overview of scRNA-seq datasets classified methods.

| Method | Geneset | Gene Numbers | Classifier |
| --- | --- | --- | --- |
| reCAT | Cyclebase | 378 | traveling salesman problem (TSP) |
| seurat | S+G2/M_marker genes(43+54) | 97 | regression |
| Torre et al. 2018 | Whitfield et al., 2002 | 874 | calculated the correlation with these genes average profile and clustered |
| Cyclone_raw | Cyclebase+GOTerm | 676 | machine learning of pairs method |
| Cyclone_merge | Cyclebase+Whitfield et al.,2002+GOTerm | 957 | machine learning of pairs method |
| Cyclum | All gene | - | autoencoder |
| Oscope | Whitfield et al., 2002 | 874 | paired-sine model |

Table S6. Cell cycle profile from scRNA-seq.

| Cell Line | Total number of cells | G1 phase number of cells (Ratio) | S phase number of cells (Ratio) | G2M phase number of cells (Ratio) | Accession |
| --- | --- | --- | --- | --- | --- |
| mESCs | 134 | 12（0.08） | 108（0.81） | 14（0.10） | 25471879 |
| mNPCs | 42 | 27（0.64） | 5（0.12） | 10（0.24） |  |
| h9ESCs | 190 | 39 (0.21) | 99 (0.52) | 52 (0.27) | 27716510 |
| hNEBs | 127 | 75 (0.59) | 37 (0.29) | 15 (0.12) |  |

Table S7. The cell cycle gene list from Hela

**G1/S phase**: ABCA7, ACD, ACYP1, ADAMTS1, ADCK2, ADCY6, ANKRD10, AP4B1, APEX2, ARGLU1, ATAD2, BAIAP2, BARD1, BDAG1, BRD7, C14orf142, C1orf63, C21orf90, C7orf23, C7orf41, C8orf38, CAPN7, CASP2, CASP8AP2, CCNE1, CCNE2, CDC25A, CDC6, CDCA7, CDCA7L, CDK20, CEP57, CHAF1A, CHAF1B, CLSPN, CREBZF, CTSD, DDX12P, DHFRL1, DIS3, DNAJB9, DNAJC3, DONSON, DSCC1, DTL, E2F1, E2F2, EIF2A, ESD, FAM105B, FAM111B, FAM122A, FANCG, FBXL20, FLAD1, FLJ41455, GINS2, GINS3, GMNN, HELLS, HORMAD1, HOXB4, HRAS, HSF2, HSPB8, INSR, INTS8, IVNS1ABP, KANK2, KCNC4, KIAA1147, KIAA1586, LENG8, LNPEP, LOC400879, LOC645561, LUC7L3, MAP2K6, MBOAT1, MCM2, MCM4, MCM5, MCM6, MDM1, MED31, MNT, MNX1, MRI1, MSH2, MZF1, NASP, NEAT1, NKTR, NPAT, NSUN5P2, NUP43, ORC1, OSBPL6, PANK2, PASK, PCDH7, PCNA, PCNAP1, PDXP, PLCXD1, PMS1, PNN, POLD3, RAB23, RECQL4, RMI2, RNF113A, RNPC3, RUNX1, SDC1, SEC62, SERPINB3, SKP2, SLBP, SLC25A27, SLC25A36, SNHG10, SPIN3, SPIN4, SRSF7, SSR3, TAF15, TIPIN, TOPBP1, TRA2A, TREX1, TRIM45, TTC14, UBR7, UHRF1, UNG, UQCC, USP53, VPS72, WDR76, WDR90, ZMYND19, ZNF141, ZNF367, ZNF414, ZNF852, ZRANB2.

**S phase**: ABCC2, ABCC5, ABHD10, ACPP, ADAM22, ANKRD18A, ANKRD36, ARHGAP42, ASF1B, ASIP, ATAD2, BBS2, BIVM, BLM, BMI1, BRCA1, BRIP1, C11orf82, C20orf111, C4BPB, C5orf42, CALD1, CALM2, CAPS, CASP2, CCDC14, CCDC150, CCDC84, CDC45, CDC7, CDCA5, CDH24, CDKN2AIP, CENPM, CENPQ, CERS6, CHML, COL7A1, COQ9, CPNE8, CREBZF, CRLS1, DCAF16, DCUN1D3, DEPDC7, DHFR, DMXL2, DNA2, DNAJB4, DNAJC6, DONSON, DSCC1, DYNC1LI2, E2F8, EFHC1, EIF4EBP2, ENOSF1, ESCO2, EXO1, EZH2, FAM178A, FANCA, FANCI, FEN1, GCLM, GOLGA6L5, GOLGA8A, GOLGA8B, GPR126, H1F0, HELLS, HIST1H2AC, HIST1H2AM, HIST1H2BC, HIST1H4B, HIST1H4C, HIST1H4H, HIST2H3A, HIST3H2A, HSPB8, IFIT1, INSIG2, INTS7, KAT2A, KAT2B, KDELC1, KIAA1598, KIFC2, LINC00339, LIPH, LMO4, LOC100288152, LOC389831, LOC400986, LYRM7, MAN1A2, MAP3K2, MASTL, MBD4, MCM8, MITF, MLF1IP, MYCBP2, MZF1, NAB1, NEAT1, NFE2L2, NRD1, NSUN3, NT5DC1, NUP160, OGT, ORC3, OSGIN2, PHIP, PHOSPHO2, PHTF1, PHTF2, PILRB, PKMYT1, POLA1, PRIM1, PRIM2, PTAR1, RAD18, RAD51, RAD51AP1, RAD54L, RBBP8, REEP1, RFC2, RHOBTB3, RHPN1, RMI1, RPA2, RRM1, RRM2, RSRC2, SAP30BP, SH3GL2, SHC1, SLC22A3, SLC25A27, SLC38A2, SP1, SRSF10, SRSF5, STAG3L1, SVIP, TMCC1, TOP2A, TRIM45, TRIM73, TTC31, TTLL7, TYMS, UBE2T, UBL3, USP1, ZBED5, ZNF217, ZWINT.

**G2 phase**: ALKBH1, ANLN, AP3D1, ARHGAP11B, ARHGAP19, ARL4A, ARMC1, ASXL1, ATL2, AURKB, BCLAF1, BORA, BRD8, BTNL9, BUB3, C12orf32, C14orf80, C15orf29, C2orf69, C5orf49, C6orf103, C9orf100, CASP3, CBX5, CCDC107, CCDC165, CCNA2, CCNF, CDC16, CDC25C, CDC42EP4, CDCA2, CDCA3, CDCA8, CDK1, CDKL5, CDKN1B, CDKN2C, CDR2, CENPL, CEP350, CFD, CFLAR, CHEK2, CIITA, CKAP2, CKAP2L, CXCL14, CYB5R2, CYTH2, DCAF7, DET1, DHX8, DNAJB1, EBI3, EMP1, ENTPD5, ESPL1, FADD, FAM110A, FAM113A, FAM72B, FAM83D, FAN1, FANCD2, FZR1, G2E3, GABPB1, GAS1, GAS2L3, GPR126, H2AFX, HAUS8, HINT3, HIPK2, HIST3H2A, HJURP, HLA-DRA, HMGB2, HN1, HP1BP3, HRSP12, HSPA2, IFNAR1, IQGAP3, KATNA1, KBTBD2, KCTD9, KDM4A, KIAA1524, KIF11, KIF20B, KIF22, KIF23, KIF5B, KIFC1, KLF6, KPNA2, LBR, LIX1L, LMNB1, LOC441052, LOC645739, LTBP3, MAD2L1, MALAT1, MELK, MEPCE, MET, MGAT2, MID1, MIS18BP1, MND1, MUC1, NBPF10, NCAPD3, NCAPH, NCOA5, NDC80, NEIL3, NFIC, NIPBL, NLRP2, NMB, NNMT, NR3C1, NUCKS1, NUMA1, NUSAP1, PIF1, PKNOX1, POLQ, PPP1R2, PSMD11, PSRC1, RANGAP1, RCCD1, RDH11, RGS3, RNF141, SAP30, SGCD, SKA3, SMC4, SORL1, SRGAP2P1, STAT1, STAT5B, STIL, STK17B, SUCLG2, SV2B, TFAP2A, TIMP1, TMEM99, TMPO, TNPO2, TOP2A, TRAIP, TRIM59, TRIM69, TRMT2A, TTC38, TTF2, TUBA1A, TUBA4A, TUBB, TUBB2A, TUBB4B, TUBD1, TYSND1, UACA, UBE2C, UBXN11, UNC5CL, VPS25, VTA1, WDR62, WISP1, WSB1, ZNF587, ZNHIT2.

**G2/M phase**: ADH4, AHI1, AKIRIN2, ANKRD40, ANLN, ANP32B, ANP32E, ARHGAP19, ARHGDIB, ARL6IP1, ASPHD2, ASXL1, ATF7IP, ATXN1L, AURKA, B4GALT1, BIRC2, BIRC5, BMP2, BUB1, C15orf23, C19orf76, C1orf96, C5orf41, C6, C9orf140, CADM1, CCDC88A, CCDC90B, CCDC99, CCNA2, CCNB2, CD97, CDC20, CDC25B, CDC27, CDC42EP1, CDCA3, CDKN2D, CENPA, CENPE, CENPF, CEP55, CFLAR, CIT, CKAP2, CKAP5, CKS1B, CKS2, CNN2, CNOT10, CNTROB, CSGALNACT1, CTCF, CTNNA1, CTNND1, DEPDC1, DEPDC1B, DIAPH3, DLGAP5, DNAJA1, DNAJB1, DR1, DUSP4, DZIP3, E2F5, ECT2, ERN2, FAM64A, FGA, FOXM1, FRZB, FYN, G2E3, GADD45A, GAS2L3, GAS6, GLI1, GOT1, GRK6, GTSE1, HCFC1, HERPUD2, HMG20B, HMGB3, HMMR, HN1, HP1BP3, HPS4, HS2ST1, HSPA13, HSPA1L, HSPA8, IDI2, IDO1, IGHA1, INADL, INPP5K, INSM1, ITPR1, KCTD2, KIAA0182, KIAA0889, KIF14, KIF20B, KIF2C, KIF5B, KLF9, KLHDC9, LBR, LMNA, LPP, LRRC17, MAPK13, MATN2, MCM4, MDC1, ME3, MIS18BP1, MKI67, MLLT4, MZT1, NCAPD2, NCOA5, NDE1, NEK2, NR5A2, NUF2, NUP35, NUP98, NUSAP1, ODF2, OIT3, OLR1, ORAOV1, PAK6, PBK, PCF11, PHF15, PIK3CD, PLAG1, PLK1, POC1A, POM121, PPP1R10, PRPSAP1, PRR11, PRR5, PSMG3, PTGER3, PTP4A1, PTPN9, PWP1, QRICH1, RAD51C, RANGAP1, RASGEF1A, RBM8A, RCAN1, RCBTB2, RERE, RNF126, RNF141, RNPS1, RRP1, SEPHS1, SEPN1, SETD8, SFPQ, SGOL2, SHCBP1, SLC17A2, SLC44A2, SMARCB1, SMARCD1, SMTN, SPAG5, SPTBN1, SRD5A1, SRF, SRSF3, SS18, STAT5B, SUV420H1, TACC3, TFF3, TGIF1, THRAP3, TLE3, TMCO4, TMEM138, TNFAIP8L1, TNPO1, TOMM34, TPX2, TRIP13, TSG101, TSKU, TSN, TTK, TUBB4B, TXNDC9, TXNRD1, UBE2D3, USP13, USP16, VANGL1, VCAM1, WIBG, WSB1, YWHAH, ZC3HC1, ZFX, ZMYM1, ZNF207, ZNF521.

**M/G1 phase**: AFAP1, AGFG1, AGPAT3, AKAP13, AMD1, ANP32E, ANTXR1, AOC2, AOC3, BAG3, BTBD3, C16orf57, C1orf96, C3orf62, C4A, CBX3, CD24, CDC42, CDK7, CDKN3, CEP70, CNIH4, CRYBA1, CTR9, CWC15, CYTH3, DCP1A, DCTN6, DEXI, DKC1, DNAJB6, DSP, DYNLL1, EIF4E, ELP3, FAM189B, FAM60A, FOPNL, FOXK2, FRS2, FXR1, G3BP1, GATA2, GDF15, GNB1, GRPEL1, GSPT1, GTF3C4, HIF1A, HIST2H2BE, HLA-DOA, HMG20B, HMGCR, HSD17B11, HSPA8, IFIT1, IFIT2, ILF2, JMJD1C, KDM5B, KIAA0182, KIAA0586, KIAA0889, KIF5B, KPNB1, KRAS, LARP1, LARP7, LNP1, LOC100507246, LOC283624, LOC91548, LRIF1, LYAR, MLLT6, MORF4L2, MRPL19, MRPS18B, MRPS2, MSL1, MTPN, NCOA3, NCS1, NFIA, NFIC, NOS1, NUCKS1, NUDT4, NUFIP2, NUP37, ODF2, OPN3, PAK1IP1, PBK, PCF11, PDGFA, PLIN3, PLK2, PPP2CA, PPP2R2A, PPP6R3, PRC1, PRR16, PSEN1, PTMS, PTTG1, RAB3A, RAD21, RAN, RHEB, RPL13A, SLC39A10, SNUPN, SRSF3, ST5, STAG1, SYNCRIP, TAF9, TCERG1, TICAM2, TLE3, TMEM138, TMEM140, TOB2, TOP1, TROAP, TSC22D1, TULP4, UBE2D3, UBE2S, USP6NL, VANGL1, VCL, VPS37C, WIPF2, WWC1, XPO4, YY1, ZBTB7A, ZCCHC10, ZNF24, ZNF281, ZNF593, ZNF98, ZNFX1, ZNFX1-AS1, ZPBP, ZSCAN5A.

Table S8. The signature gene list of cell type.

**ESC specific genes**: RRM2, MRPL13, XRCC5, FGFR1, BUB3, NUP107, SET, PARP1, HSPA9, RPL22, FGFR1, MTF2, NAP1L1, XRCC5, PARP1, NAP1L1, FGFR1, CSE1L, HSPA9, FGFR1, SNRPD1, GEMIN6, HSPE1, NAP1L1, BUB3, HSPA9, FGFR1, CSE1L, NAP1L1, DHX9, DARS2, BUB3, SMC2, HSPA9, BLM, CDKN3, BUB3, NONO, NAP1L1, NUP107, PLK4, RRM2, BLM, MSH2, NUSAP1, CDKN3, CSE1L, RRM1, BUB1, DARS2, HSPA9, SMC2, BUB3, EEF1E1, MRPL13, HSPA9, CBX3, NONO, NUP107, XRCC5, RRM1, DHX9, FGFR1, PABPC1, BUB3, MTF2, CBX3, CSE1L, NAP1L1, IPO9, XRCC5, BLM, HSPA9, CSE1L, FGFR1, DHX9, NONO, BUB3, SMC2, NAP1L1, HSPA9, XRCC5, CBX3, PABPC1, NONO, DHX9, MTF2, CSE1L, RPS23, PRIM2, RRM2, CBX3, MSH2, POLE2, NAP1L1, NUSAP1, HSPE1, BLM, PRMT3, CDKN3, PLK4, CSE1L, SMC2, RPL22 , PARP1, XRCC5, UQCRH, RPL22, RRM1, BLM, RRM2, BUB1, SMC2, MSH2, PRIM2, XRCC5, NUSAP1, DARS2, MRPL13, POLE2, NUP107, IPO9, PLK4, SNRPD1, DHX9, MTF2, BUB3, CBX3, CDKN3, CSE1L, CBX3, HSPE1, BUB1, NUSAP1, DARS2, KPNA2, BUB3, RRM2, CSE1L, PLK4, HSPA9, MTF2, MRPL13, IPO9, PARP1, CDKN3, KPNA2, CSE1L, RRM2, DARS2, PRMT3, RPS23, HSPA9, PABPC1, HSPE1, MSH2, DHX9, SNRPD1, CBX3, SET, RPL22, CSE1L, CDKN3, KPNA2, NUSAP1, CBX3, BUB1, DARS2, RRM2, MTF2, RRM1, CSE1L, KPNA6, HSPA9, NONO, SET, FGFR1, CBX3, NAP1L1, MRPL13, BUB3, RRM2, NONO, FGFR1, SET, HSPA9, GEMIN6, SNRPD1, DHX9, CSE1L, PABPC1, NUP107, MTF2, GEMIN6, HSPA9, NONO, FGFR1, RPL22, DHX9, CSE1L, KPNA2, CBX3, PLK4, BUB1, RRM2, BLM, HSPA9, PABPC1, HSPE1, SMC2, SQLE, NUSAP1, RPA3, CSE1L, SQLE, NAP1L1, FGFR1, NAP1L1, NONO, HSPA9, PABPC1, RPL22, NUSAP1, CBX3, SMC2, PLK4, KPNA6, MSH2, BLM, UQCRH, CDKN3, PRIM2, HSPA9, CSE1L, NUP107, DHX9, MRPL13, RRM2, BUB3, SNRPD1, XRCC5, SET, RPS23, RRM1, PABPC1, SMC2, CBX3, HSPA9, BUB1, MRPL13, HSPE1, SQLE, RPL22, RRM1, STIP1, PRMT1, RUVBL2, MRPL37, NDUFS2, SEPHS2, ENO1, PRMT1, ERP29, NME2, BAX, SNRPA, STIP1, PRMT1, FARSA, NDUFA11, SNRPA, NDUFS2, RPL13, RUVBL2, CRABP2, PDHA1, MRPL37, CCND1, LSM4, MID1IP1, CDC20, NDUFS2, PLK1, LSM4, TGIF2, NDUFS2, CRABP2, PPP4C, KRAS, TGIF2, PHB, LSM4, NME2, PRMT1, BIRC5, ETFA, SNRPA, POP7, STIP1, TIMM13, ENO1, RNPS1, CCND1, NTHL1, PHB, CCND1, MID1IP1, EEF2, CCND1, BAX, CRABP2, MID1IP1, ENO1, BAX, PDHA1, ERP29, CRABP2, KRAS, PHB, PDHA1, NDUFS2, BIRC5, RCN2, EIF4EBP1, RNPS1, CCND1, CRABP2, KRAS, CRABP2, C11orf48, RCN2, NME2, NDUFS2, FARSA, NDUFA11, CCND1, KRAS, NME2, ERP29, PRMT1, FARSA, RUVBL2, SNRPA, STIP1, CDC20, FARSA, PRMT1, POP7, TIMM13, ERP29, BIRC5, BAX, ERP29, TIMM13, STIP1, SNRPA, POP7, ETFA, EEF2, NTHL1, STIP1, POP7, PRMT1, ETFA, CDC20, SNRPA, MID1IP1, RUVBL2, PDHA1, EIF4EBP1.

**Stem cell population maintenance:** ABL1, ARHGEF2, ARID1A, ASCL2, ASPM, BCL9, BCL9L, BMP7, BMPR1A, BRAF, CDC73, CDH2, CDX2, CNOT1, CNOT2, CNOT3, CTC1, CTNNA1, CTNNB1, CTR9, CUL4A, CX3CL1, DDX6, DIS3L2, DLL1, DOCK7, DPPA4, DRD2, EIF4E, EIF4ENIF1, ELAVL1, ELF5, EOMES, EPHA1, ESRRB, FANCC, FANCD2, FERMT1, FGF10, FGF13, FGF2, FGF4, FOXC1, FOXD3, FOXO1, FOXO3, FOXP1, FUT10, FZD7, GATA2, GATA3, GNL3, HES1, HES5, HMGA2, HNF1B, HNRNPU, HOOK3, HTR2B, IGF2BP1, JAG1, KDM2B, KDM3A, KDM4C, KIT, KLF10, KLF4, LBH, LDB1, LDB2, LEO1, LIF, LIG4, LIN28A, LOXL2, LSM1, MAPK1, MCPH1, MED10, MED12, MED14, MED15, MED21, MED27, MED28, MED30, MED7, METTL14, METTL3, MMP24, MTF2, MYOCD, NANOG, NANOS2, NCOA3, NDE1, NIPBL, NKAP, NODAL, NOG, NOTCH1, NOTCH2, NR2E1, PADI4, PAF1, PAFAH1B1, PAX2, PAX6, PAX8, PBX1, PCM1, PELO, PIWIL2, POU5F1, PRDM14, PRDM16, PROX1, PRRX1, PTPRC, RAB10, RAF1, RBPJ, RIF1, RTF1, SALL1, SALL4, SAV1, SETD6, SFRP1, SHH, SIRT6, SIX2, SKI, SMAD2, SMAD4, SMC1A, SMC3, SMO, SOX2, SOX4, SOX9, SPI1, SRF, SRRT, SS18, STAT3, TAL1, TBX3, TDGF1, TERT, TET1, TGFB1, TP63, TRIM8, TUT4, VANGL2, VPS72, WNT5A, WNT7A, WNT7B, WNT9B, XRCC5, YAP1, ZC3H13, ZFP36L2, ZHX2, ZIC3, ZNF322, ZNF358, ZNF706, ZSCAN10.

**Neuroblast proliferation:** ARTN, ASCL1, ASPM, CTNNA1, CTNNB1, CX3CL1, CX3CR1, DAGLA, DAGLB, DCT, DISC1, DMRTA2, DOCK7, DRD2, EML1, FGF13, FGFR1, FGFR2, FRS2, FZD3, FZD9, GLI3, HHIP, HIF1A, ID4, KCNA1, KCTD11, LEF1, LRRK2, NDE1, NEUROD4, NF1, NOTCH1, NUMB, NUMBL, OTP, PAFAH1B1, PAX6, PLXNB2, RAB10, RACGAP1, SHH, SIX3, SMARCD3, SMO, SOX10, SOX5, TEAD3, TGFB1, VAX1, VEGFA, VEGFC, WNT3A, ZNF335.

**Mitotic cell cycle arrest**: CDC14C, CDC14B, GADD45A, DUSP1, CDKN1A, CDKN1B, FAP, E4F1, MAGI2, MCPH1, GADD45GIP1, PNPT1, NKX3-1, RGCC, CDC14A.

**Neuron development:** HTRA2, HS6ST1, PITX3, TDP2, GLI2, NR2F6, SRF, SKI, SKIL, LIF, XBP1, EN2, POU3F2, CNTF, PBX3, MAPK8, MAPK9, DGKG, PSEN1, ASCL1, PPT1, MAPK10, EPHA5, TGFB2, SKOR1, EN1, MEF2C, NEUROD1, MTMR2, DPYSL3, BECN1, IHH, NEUROD2, CTF1, SKOR2, CCSAP, TENM4, MFSD8, THOC2, FIG4, ISL2, SECISBP2, WNK1, NEUROD4, TENM2, ATP8A2, VSX1, TENM3, TENM1, MYT1L.

**Neuron differentiation**:, UNC119B, WNT10B, WNT9A, WNT9B, SNPH, DAPK3, LMX1B, FZD7, ALDH1A2, WNT11, WNT2, HSPA5, EDN3, CEBPB, POU3F2, S1PR1, PTPRD, SMARCA1, EPHA2, HOXC8, RYK, SOX11, PPP1CC, ID1, WNT5A, CASP3, ASCL1, NAPA, WNT3, WNT3A, WNT4, WNT7B, MTPN, COPS2, PPIA, CDK5, ATP2B2, ID3, MAP2K1, EMX2, MEF2C, POU4F2, UNC119, FZD5, PIN1, RUNX2, FZD2, CDK5R1, NNAT, NTRK2, PROX2, ADNP2, PAPD4, LDB1, HIPK1, BRSK2, NLGN4X, GPC2, DCLK2, PCSK9, BRSK1, PROX1, NEUROG1, WNT2B, WNT8B, PIGT, TRAPPC9, IER2, WNT10A, NIF3L1, HOXD1, XRN2, WNT8A, WNT5B, NEUROG2, HIPK2, FZD8, NGRN, FZD3, ITM2C, NRBP2, DDIT4, BTG4, BARHL2, IL1RAPL1, MYEF2, WNT16, MBD1, HDAC9, TRPC5, MYT1L, FZD4, FZD10, FZD1, CNTN6, HDAC5, TRPC6, WNT6, DNMT3A.

**Cell fate specification:**, TBX1, TBX6, ITGB1, POU1F1, CTNNB1, NOTCH1, SOX9, PSEN1, FKBP8, IHH, SHH, CDON, C8orf22, SMO, PRDM14, PPDPF, FOXA2.

Table S9. qPCR primer sequences.

| Primer | Primer Sequence(5'to3') | length(bp) |
| --- | --- | --- |
| Gas2l3_For  Gas2l3_Rev  Ckap2l_For  Ckap2l_Rev  Fam83d_For  Fam83d_Rev Ndc80_For Ndc80_Rev Nuf2_For  Nuf2_Rev Cenpi_For Cenpi_Rev | GAGATTGGTCGGATTGTGTC  CGAGGCATTAAGCAAGGTC  ATTCCAGACTAAACCAGCCAG AATGACACTGCTCTAGGTTGC AGACATCGACATCTTCCGAG  CCTGTAATTGTCCGAACTGTC  AGTACACACAAACCGACATCG TGTCATTAAGTGGTCTTGGGTC CCGGCTGGAGCACTTCTAC  CAAATGGGCATAAAGGAATC CAGTAATAGCCGTCAAACCAG CACCTTCAGTTCGATCATCAG | 20  19  21  21  20  21  21  22  19  20  21 |

For:forward strand, Rev: reverse strand
